# Enhancer Associated Long Non-coding RNA Transcription and Gene Regulation in Experimental Models of Rickettsial Infection

**DOI:** 10.3389/fimmu.2018.03014

**Published:** 2019-01-09

**Authors:** Imran H. Chowdhury, Hema P. Narra, Abha Sahni, Kamil Khanipov, Yuriy Fofanov, Sanjeev K. Sahni

**Affiliations:** ^1^Department of Pathology, University of Texas Medical Branch, Galveston, TX, United States; ^2^Institute for Human Infections and Immunity, University of Texas Medical Branch, University Boulevard, Galveston, TX, United States; ^3^Department of Pharmacology, University of Texas Medical Branch, University Boulevard, Galveston, TX, United States

**Keywords:** *Rickettsia*, long non-coding (lnc) RNA, enhancer long non-coding (elnc) RNA, RNA sequencing, transcription start site, inhibitor of DNA binding 2 protein, apolipoprotein L 10b, host immune responses

## Abstract

Recent discovery that much of the mammalian genome does not encode protein-coding genes (PCGs) has brought widespread attention to long noncoding RNAs (lncRNAs) as a novel layer of biological regulation. Enhancer lnc (elnc) RNAs from the enhancer regions of the genome carry the capacity to regulate PCGs in *cis* or in *trans*. Spotted fever rickettsioses represent the consequence of host infection with Gram-negative, obligate intracellular bacteria in the Genus *Rickettsia*. Despite being implicated in the pathways of infection and inflammation, the roles of lncRNAs in host response to *Rickettsia* species have remained a mystery. We have profiled the expression of host lncRNAs during infection of susceptible mice with *R. conorii* as a model closely mimicking the pathogenesis of human spotted fever rickettsioses. RNA sequencing on the lungs of infected hosts yielded reads mapping to 74,964 non-coding RNAs, 206 and 277 of which were determined to be significantly up- and down-regulated, respectively, in comparison to uninfected controls. Following removal of short non-coding RNAs and ambiguous transcripts, remaining transcripts underwent in-depth analysis of mouse lung epigenetic signatures H3K4Me1 and H3K4Me3, active transcript markers (POLR2A, p300, CTCF), and DNaseI hypersensitivity sites to identify two potentially active and highly up-regulated elncRNAs NONMMUT013718 and NONMMUT024103. Using Hi-3C sequencing resource, we further determined that genomic loci of NONMMUT013718 and NONMMUT024103 might interact with and regulate the expression of nearby PCGs, namely Id2 (inhibitor of DNA binding 2) and Apol10b (apolipoprotein 10b), respectively. Heterologous reporter assays confirmed the activity of elncRNAs as the inducers of their predicted PCGs. In the lungs of infected mice, expression of both elncRNAs and their targets was significantly higher than mock-infected controls. Induced expression of NONMMUT013718/Id2 in murine macrophages and NONMMUT024103/Apol10b in endothelial cells was also clearly evident during *R. conorii* infection *in vitro*. Finally, shRNA mediated knock-down of NONMMUT013718 and NONMMUT024103 elncRNAs resulted in reduced expression of endogenous Id2 and Apl10b, demonstrating the regulatory roles of these elncRNAs on their target PCGs. Our results provide very first experimental evidence suggesting altered expression of pulmonary lncRNAs and elncRNA-mediated regulation of PCGs involved in immunity and during host interactions with pathogenic rickettsiae.

## Introduction

Arthropod-borne *Rickettsia* species include obligate intracellular, Gram-negative bacteria known to cause spotted fever and typhus groups of rickettsial dieases in humans (1). The clinical spectrum of spotted fever group (SFG) rickettsioses varies in severity from mild to fatal cases of Rocky Mountain spotted fever (RMSF) caused by *Rickettsia rickettsii*, Mediterranean spotted fever (MSF) due to *R. conorii*, and Queensland tick typhus following infection with *R. australis* (1). A majority of human rickettsial diseases involve transmission from arthropod vectors, for example naturally circulating infected ticks in case of *R. rickettsii* and *R. conorii*. Due mainly to the predilection of pathogenic rickettsiae to target endothelial cells lining the microvasculature in their mammalian hosts and cell-to-cell spread during the course of infection (1, 2), a prominent feature of pathogenesis is the innate immune activation and inflammatory perturbations of microvascular endothelium, leading to complications such as ocular inflammation or retinitis, myocarditis, endocarditis, pulmonary, and cerebral edema due to fluid imbalance associated with the derangements of endothelial barrier, and multi-organ failure in severe cases (1–6). Employing both patient samples and established experimental models of infection, a number of studies have delved into the definition of host responses during rickettsial infections (7, 8), but the mechanisms underlying the activation and regulation of such immune mechanisms remain largely unknown.

Functional annotation of the mammalian genome (FANTOM) and Encyclopedia of DNA Elements (ENCODE) projects have challenged the central dogma of molecular biology by suggesting that non-protein-coding regions carry multiple overlapping codes that profoundly affect gene expression and other cellular processes. Notably, protein-coding sequences occupy <2% of the genome in mammals, whereas a much larger fraction is transcribed into non-coding RNAs (ncRNAs) (9–12). A majority of ncRNA transcripts are functionally active RNAs broadly classified into short non-coding RNAs (miRNA) of less than and long non-coding (lnc) RNAs of more than 200 nucleotides (13, 14). Short ncRNAs are now established as highly versatile molecules capable of interacting with other RNAs, DNA, or a vast repertoire of proteins, highlighting their regulatory potential (15). In recent years, lncRNAs have also been implicated in diverse major biological processes, including immune regulation, cell cycle, apoptosis, post-transcriptional, and translational regulation, epigenetic modification, and nuclear genome organization, highlighting their regulatory activities in the determination of host-pathogen interactions (12). Among these, an important sub-class of lncRNAs derived from the enhancer loci of the genome are designated as enhancer long non-coding (elnc) RNAs or eRNAs (16–19). Active enhancers are traditionally considered as the principal regulatory components of the genome capable of enabling cell or tissue type and cell-cycle specific gene expression in *cis* and *trans*. As such, elncRNA(s) have received considerable attention by virtue of their ability to control protein coding genes (PCGs) by locus control mechanism (20). Potential elncRNAs are generally characterized by higher occupancy of chromatin monomethylation of histone H3 at lysine 4 (H3K4Me1) (signature of enhancer loci) when compared to trimethylation of histone H3 at lysine 4 (H3K4Me3) (signature of promoter loci) and other epigenetic signatures such as RNA pol II, DNaseI hypersensitivity site, and p300 binding sites at or around the transcription start site (TSS). Enhancer elements in the genome play an active role in controlling the transcription of PCGs by stabilizing enhancer-promoter interactions (16, 21).

Although NCBI chromatin and epigenetic gene expression omnibus (GEO) databases have enabled the identification of cell- and tissue-specific active enhancers in both human and mouse (22, 23), active elncRNAs are currently characterized in only a limited numbers of cells and tissues and their functional roles in the host responses and pathogenesis of rickettsial diseases remain poorly understood. In the present study, we have elucidated lncRNA signatures of the host lungs in a murine model of rickettsial disease and identified two elncRNAs that may be involved in the host response to infection.

## Materials and Methods

### Preparation of *R. conorii* Stocks

Monolayers of cultured Vero cells as the host were infected with *R. conorii* (Strain Malish 7) to allow for intracellular growth and replication of rickettsiae. Heavily-infected cells (infection of ≥80% of cells with ≥50 intracellular rickettsiae) were gently lysed using glass beads for the isolation and purification of rickettsial stocks by differential centrifugation. The rickettsial preparations were stored at −80°C by slow freezing as aliquots of ≤ 0.5 ml and gently thawed on ice to avoid loss of viability. The infectious titer of stocks thus prepared was determined by rickettsial citrate synthase (gltA)-based quantitative PCR and plaque formation assays using standard protocols and procedures (24, 25).

### Mice and Infection

To identify lncRNA transcripts expressed during *R. conorii* infection, we employed an established mouse model of infection (26). C3H/HeN mice were obtained from the Jackson Laboratory and housed in an ABSL3 laboratory suite. Following acclimatization, the animals were infected with a high dose of *R. conorii* (2.25 × 10^5^ pfu/mouse) administered through the tail vein injection. The control group of animals received identical volume of saline intravenously (26). The animals were then monitored at least once daily for overt signs of disease (ruffled fur, hunched posture, and photophobia) and the body weights were recorded. On day 3 post-infection, mice were euthanized and the lungs were removed aseptically. The tissues thus collected were either snap-frozen or stored at −20°C in RNAlater™ solution. All the animal procedures were performed in accordance to the National Institutes of Health Guide for the Care and use of Laboratory Animals, and were maintained by the approval of Institutional Animal Care and Use Committee at the University of Texas Medical Branch (UTMB) (protocol #1109042). The University has a file with the Office of Laboratory Animal Welfare and an approved Assurance Statement (#A3314-01). Use of any cell line in this study was exempt by Institutional Review Board (IRB), and approved by Institutional Biosafety committee (IBC), UTMB.

### RNA Extraction and cDNA Library Preparation

Total RNA from lung tissues was extracted using TRIzol reagent according to the manufacturer's instructions (Invitrogen). RNA samples were subjected to treatment with DNaseI (NEB) to remove any contaminating DNA and then enriched with Ribo-Zero rRNA Removal kit (Illumina). Concentration of RNA in sample preparations using the MultiSkan Go UV/Vis instrument for microsample analysis (Thermo Scientific) and the quality of RNA was evaluated on a bioanalyzer (Agilent Technologies). The samples with an RNA integrity number (RIN) of >9 were subjected to RNA-sequencing (12). Briefly, RNA fragments of 50 bases were generated by incubating purified total RNA in a fragmentation buffer (Ambion) and fragmented RNA was then ligated with 5′ and 3′-adaptors using a T4 RNA ligase (NEB). Adaptor-ligated RNAs were reverse transcribed and subjected to PCR amplification with barcoded primers (Illumina) (27, 28). Finally, amplified cDNA libraries were purified using standard gel purification procedures.

### RNA Sequencing, Mapping, and Data Analysis

RNA sequencing was performed on an Illumina HiSeq 1500 at the Next Generation Sequencing Core facility at the UTMB. Briefly, 50 base long reads were obtained from the RNA derived from the lungs of *R. conorii-*infected and uninfected control (*n* = 3 for each) mice. The first 14 bases of the reads were trimmed and only reads with high base quality (phred score >15) were used for downstream analysis. All high quality reads were then grouped according to their designation as infected or control. To identify ncRNAs, all reads were first mapped to *Mus musculus* Ref-seq (mm9) genome (to remove reads from mRNAs), and the remaining unmapped reads were then mapped to known mouse ncRNA transcripts in the NONCODE (NONCODE_V4) database with an allowance of up to two mismatches employing CLC Genomic Workbench 9.0.1 (http://www.clcbio.com) RNA-sequencing Analysis tool. The RNA-sequencing data were normalized by calculating “reads per kilobase million” (RKPM) as described earlier (12). Expression of all mRNA and ncRNA transcripts was determined in each infected sample by dividing the normalized reads from *R. conorii-*infected sample with those from the corresponding mock-infected sample. Mann–Whitney *U*-Test was used to compare the differences in relative abundance of identified lncRNA and mRNA transcripts between groups. We next applied Min/Max method to identify the expression of potential lncRNA candidates and their nearby PCGs. Up-regulation of lncRNA transcripts and/or PCGs was determined as the ratio of the lowest normalized reads in the infected group to the highest normalized reads in the control group (*n* = 3). Conversely, down-regulation of lncRNA transcripts and/or PCGs was ascertained by dividing the highest normalized reads in the infected group with the lowest normalized reads of the sample in the uninfected control group (*n* = 3 for each group). The FASTQ files for RNA sequencing data were submitted to GenBank (Accession number GSE121808).

### Quantitative Real-Time PCR (qRT-PCR)

Approximately 1 μg of RNA from mock and *R. conorii-*infected lungs was reverse transcribed using a cDNA Synthesis Kit (Applied Biosystems). The cDNA was subjected to qPCR using SYBR Green as the reporter on a StepOnePlus instrument (Applied Biosystems). PCR reactions were performed in triplicate using the primer sequences listed in Supplementary Table 3. The datasets were normalized using 18S RNA as the housekeeping gene. The levels of expression and relative quantification were determined via calculations based on the 2^−ΔΔ*Ct*^ method (12).

### Cataloging of lncRNAs

To catalog lncRNAs, we captured the strand of origin, nature of origin, chromosomal origin, number of exons, and lengths of all the differentially expressed lncRNAs from NONCODE_V4 database. We grouped lncRNAs based on their strand of origin (sense or anti-sense), source of origin (chromosome number 1–20 and mitochondrial DNA), nature of origin [sense-exonic, sense intronic, antisense, antisense-exonic, antisense intronic and LINC (long intergenic non-coding) RNA], exonic composition (uni-exonic, bi-exonic and multi-exonic), and the length of transcripts (length ~200–500, 501–2,000, 2,001–5,000, and ≥5,001 bp) as described earlier (12).

### TSS Evidence and Filtering of Up-Regulated lncRNA Transcripts

We utilized UCSC genome browser to further categorize up-regulated lncRNA transcripts based on their origin and orientation to the nearby PCGs. We cataloged them into “head to head,” “head to tail,” “tail to tail,” and “tail to head” orientation, and all these classifications were utilized for selection of transcripts for further downstream analysis (29).

The TSSs of lncRNAs and nearby PCGs as reported in the NONCODE database and UCSC genome browser (www.genome.ucsc.edu), respectively, were used to compute the distance of lncRNAs to the nearest PCGs for downstream filtering of transcripts. To identify up-regulated elncRNAs, lncRNA transcripts originating from sense-exonic, sense intronic, antisense to the PCGs, antisense-exonic, antisense intronic and LINC transcripts for which TSS are within a 2 kb window of TSS or transcription end site (TES) of nearby PCGs (29, 30), were excluded from the analysis. The remaining lncRNA transcripts were analyzed for chromatin and epigenetic signatures as described below.

### Analysis of ChIP-Seq GEO Data for Chromatin and Epigenetic Signatures

We performed quantitative assessment of chromatin signatures H3K4me1 and H3K4me3 around the TSS of up-regulated lncRNAs to identify elncRNAs. Briefly, ChIP-Seq datasets for H3K4me1 (GSM769013) and H3K4me3 (GSM769012) in the mouse lung were downloaded from the NCBI GEO database (http://www.ncbi.nlm.nih.gov/gds). The genomic sequence of 4 kb around the TSS of filtered lncRNAs was extracted and mapped to the ChIP-seq datasets using Samtools. The reads were normalized by subtracting the reads of lung input (GSM 918739) within same 4 kb window. Based on the profile of chromatin signatures around the TSS of lncRNAs, we cataloged them into either enhancer or promoter (or canonical) lncRNAs. To identify active elncRNAs in the mouse lung, we utilized GEO data for RNA Pol II (GSM918724), p300 co-activator binding site (GSM722862), DNaseI hypersensitivity site (GSM1014194), and CTCF (CCCTC binding protein) binding sites (GSM918722). The mapping of reads was performed with an allowance of upto two base mismatches, and all reads mapping within the 4 kb window around the TSS were used for identification of active elncRNAs as described earlier (31).

### Analysis of Hi-3C GEO Data and qRT-PCR

To determine the interaction(s) between an active elncRNA and its nearby PCGs, we applied a combinatorial approach based on the analysis of high throughput sequencing of Chromosome Conformation Capture (Hi-C) and virtual 4C profiles by 3D Epigenome browser (www.3dgenome.org). We uploaded the Hi-C tracks to identify the signals for interactions between the coordinates of elncRNAs and potential PCGs. For further confirmation, we utilized visualization of virtual 4C profiles to identify the location of genomic contact loci of elncRNA in relation to the anchoring point for the promoter of nearby PCGs (32, 33). We next performed qRT-PCR for active elncRNAs and their targets on the RNA from infected mouse lungs to investigate the possibility of correlative changes in their expression. The primer sequences for qRT-PCR are listed in Supplementary Table 3.

### Cell Culture and Infection

Murine RAW264.7 macrophages, NIH3T3 fibroblasts, and SV40-transformed mouse endothelial cells (SVEC) 4-10 were maintained at 37°C with 5% CO_2_ in Dulbecco's Modified Eagle's Medium supplemented with 10% fetal bovine serum (Aleken Biologicals), 10 mM L-glutamine (Thermo Fisher Scientific), 100 Units each of Penicillin and Streptomycin according to previously published procedures (34–36). For experiments with rickettsiae, the Penicillin-Streptomycin mix was removed from the culture medium a minimum of 24 h prior to and during the infection. All experiments were performed with exponentially growing cells at relatively low passage numbers of 5–15. Because endothelial cells and macrophages are the major targets of rickettsial infection, we infected RAW264.7 macrophages and SVECs with *R. conorii* for 3 and 24 h (MOI = 5).

### Reporter Constructs and Transient Transfection

Genomic loci of active elncRNAs flanking the boundaries of epigenetic signature were PCR amplified using Phusion High Fedelity DNA polymerase (NEB). The purified PCR fragments were cloned in both sense and antisense orientation upstream of the SV40 promoter in a pGL3 firefly promoter plasmid (Promega). Simultaneously, we picked two other genomic coordinates flanking Chr18: 60429728-60430126 and Chr13: 60430551-60430958 with a minimal ratio of H3K4Me1/H3K4Me3 and negligible peaks of other epigenetic signatures (RNA pol II, P300, and DNaseI hypersensitivity site) and cloned them into the pGL3 promoter plasmid to serve as negative controls in our experiments. All inserts in the promoter plasmid were confirmed by DNA sequencing at the UTMB Molecular Genomics Core facility. The primer sequences and restriction sites of inserts are also listed in Supplementary Table 3. Transfection-grade, endotoxin-free plasmids were prepared using an EndoFree® Plasmid Purification kit (Qiagen). We transfected these plasmids along with a pRL-SV40 plasmid as an internal control in mouse NIH3T3 fibroblasts and RAW264.7 macrophages at about 80% confluence. For each assay, 1 μg of blank plasmid (pGL3 promoter plasmid) or elncRNA constructs or negative controls, and 200 ng of pRL-SV40 were co-transfected using Lipofactamine 3000 (Invitrogen). After 24 h of transfection, cell lysates were prepared and dual luciferase assay was performed according to manufacturer's instructions (Promega). Firefly and Renilla luciferase signals were recorded using a GloMax® 20/20 Single-Tube Luminometer (Promega). The signal ratio in each well was calculated by dividing the luciferase signal by Renilla signal.

### Knock-Down of elncRNAs and qRT-PCR

Two distinct targets based on the published guidelines (37) were chosen to design short hairpin RNAs (shRNAs) for elncRNA knock-down using an RNAi consortium designing tool (www. broadinstitute.org). To avoid potential confounding effects of non-specific knock-down, shRNA sequences were further verified through a BLAST search in the NCBI. The shRNAs were then cloned into a pLKO.1 lentivirus puro vector (Addgene plasmid #8453; Addgene, Cambridge, MA, USA), followed by sequencing at the UTMB sequencing core to confirm the orientation of the insert. The shRNA target sites and sequences are listed in the Supplementary File 1 and Supplementary Table 3. Endotoxin-free plasmid preparations and transfection of plasmids carrying shRNA hairpin constructs or scrambled sequences (control) were carried out as detailed above. Transfected cells were allowed to recover for 24 h prior to infection with *R. conorii*. Efficiency of knock-down was confirmed by qPCR assay.

### Statistical Analysis

D'Agostino & Pearson omnibus normality test was performed to ensure normal distribution of data. Comparisons between the unmatched groups were done by unpaired *t*-test or Mann–Whitney *U*-test, whereas comparisons amongst the matched groups were performed by paired *t-*test or Wilcoxon signed rank test. The correlative analysis was performed using Spearmann correlation test. GraphPad Prism version 5 (GraphPad Software Inc., San Diego, California) was used for all statistical analyses with *P* ≤ 0.05 suggesting statistically significant changes.

## Results

**Figure 1:**
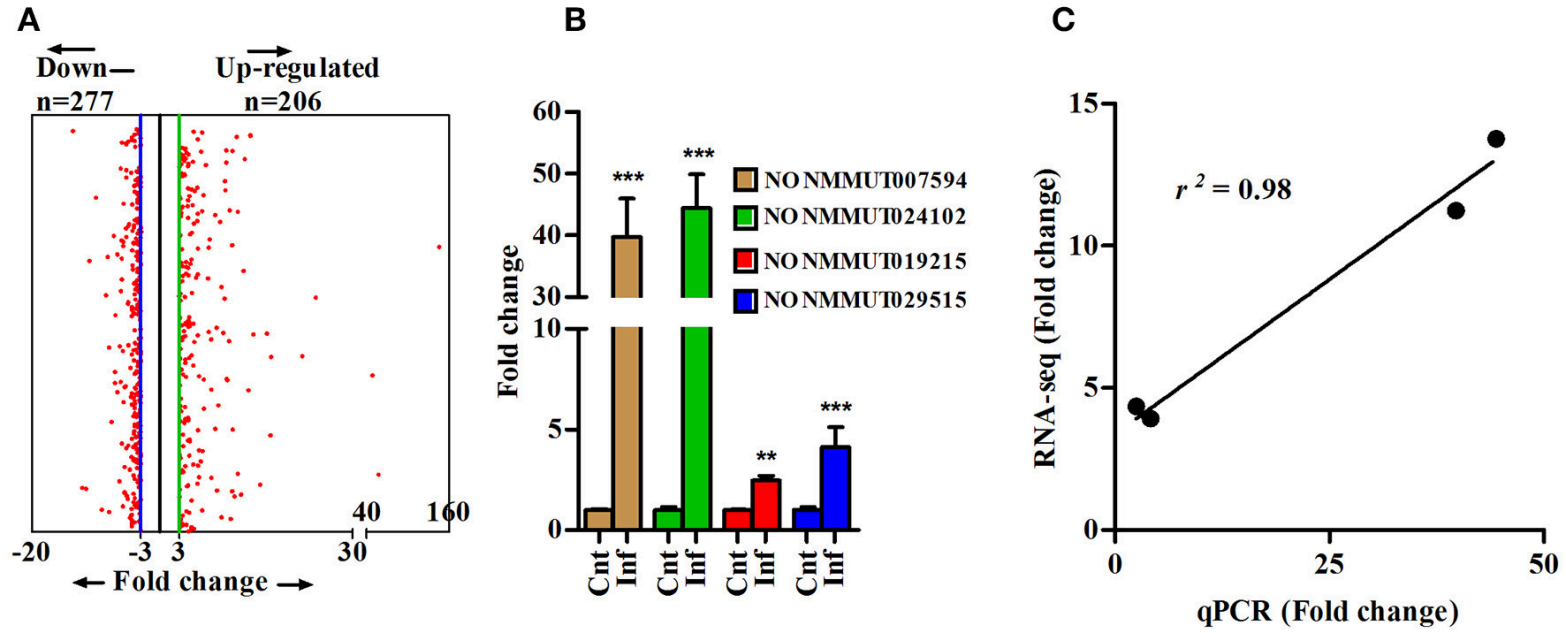
Differentially expressed lncRNA transcripts in lungs of mouse infected with *R. conorii*. **(A)** Differentially expressed lncRNA transcripts (*n* = 3, control and infected mice) based on Min/Max method (3-fold cut-off); **(B)** Validation of expression of four randomly selected up-regulated lncRNA transcripts (from RNA-sequencing analysis) by qPCR in infected mouse lungs (*n* = 4). The error bars represents standard error of mean (SEM) and the level of significance are shown as **P ≤ 0.01, and ***P ≤ 0.001; and **(C)** Spearmann correlation of fold change of 4 lncRNA transcripts as determined by RNA sequencing and qPCR methods (*r*^2^ = 0.98, *P* < 0.001).

To determine changes in the lncRNA profile during rickettsial infection, we performed RNA sequencing (RNA-seq) on the lungs of susceptible mice infected with *R. conorii* on day 3 post-infection. The step-by-step schematics for the methods utilized and decision points are presented in Supplementary Figure 1. A total of 152.46 (*n* = 3) and 160.3 (*n* = 3) million reads were obtained from the lungs of mock- and *R. conorii-*infected mice, respectively. We first mapped the libraries to the Ref-Seq to remove reads originating from the annotated *Mus musculus* (mm9) coding transcripts and the remaining 106.71 and 108.08 million reads were then mapped to NONCODE_V4 database containing 74,964 ncRNA transcripts (38). The total number of reads from each cDNA library and the reads mapping to mRNAs and ncRNAs are presented in Supplementary Table 1. As expected, the reads mapping neither to Ref-seq genes nor ncRNAs predominantly corresponded to the ribosomal RNA (rRNA) and transfer RNA (tRNA) transcripts. All coding and ncRNA transcripts with undetectable expression were excluded from the analysis. We thus identified a total of 1,168 and 6,216 ncRNAs that were either up- or down-regulated, respectively, in the lungs of infected mice (Cut-off fold ≥3, *P* ≤ 0.05) (Supplementary Figure 2). We next applied Min/Max method to identify ncRNA transcripts exhibiting a high degree of regulation in response to infection, restricting the number of up- and down-regulated ncRNAs in our datasets to 206 and 277, respectively, of which further removal of any ncRNA transcripts with a length of <200 bp allowed us to retain 179 up-regulated and 271 down-regulated lncRNA transcripts (Figure 1A). To validate our results from RNA-seq, we randomly selected 4 up-regulated ncRNA transcripts (NONMMUT007594, NONMMUT019215, NONMMUT024102, and NONMMUT029515) to independently determine their expression levels by qRT-PCR. Notably, the expression of all of these transcripts in the lungs of *R. conorii*-infected mice was significantly higher, albeit to varying degrees in terms of average fold-induction, than the corresponding controls (Figure 1B). Finally, comparison of our data from RNA sequencing and qRT-PCR using Spearmann correlation analysis revealed high level of correlation, confirming excellent agreement between the findings from two independent approaches (*r*^2^ = 0.98) (Figure 1C).

**Figure 2:**
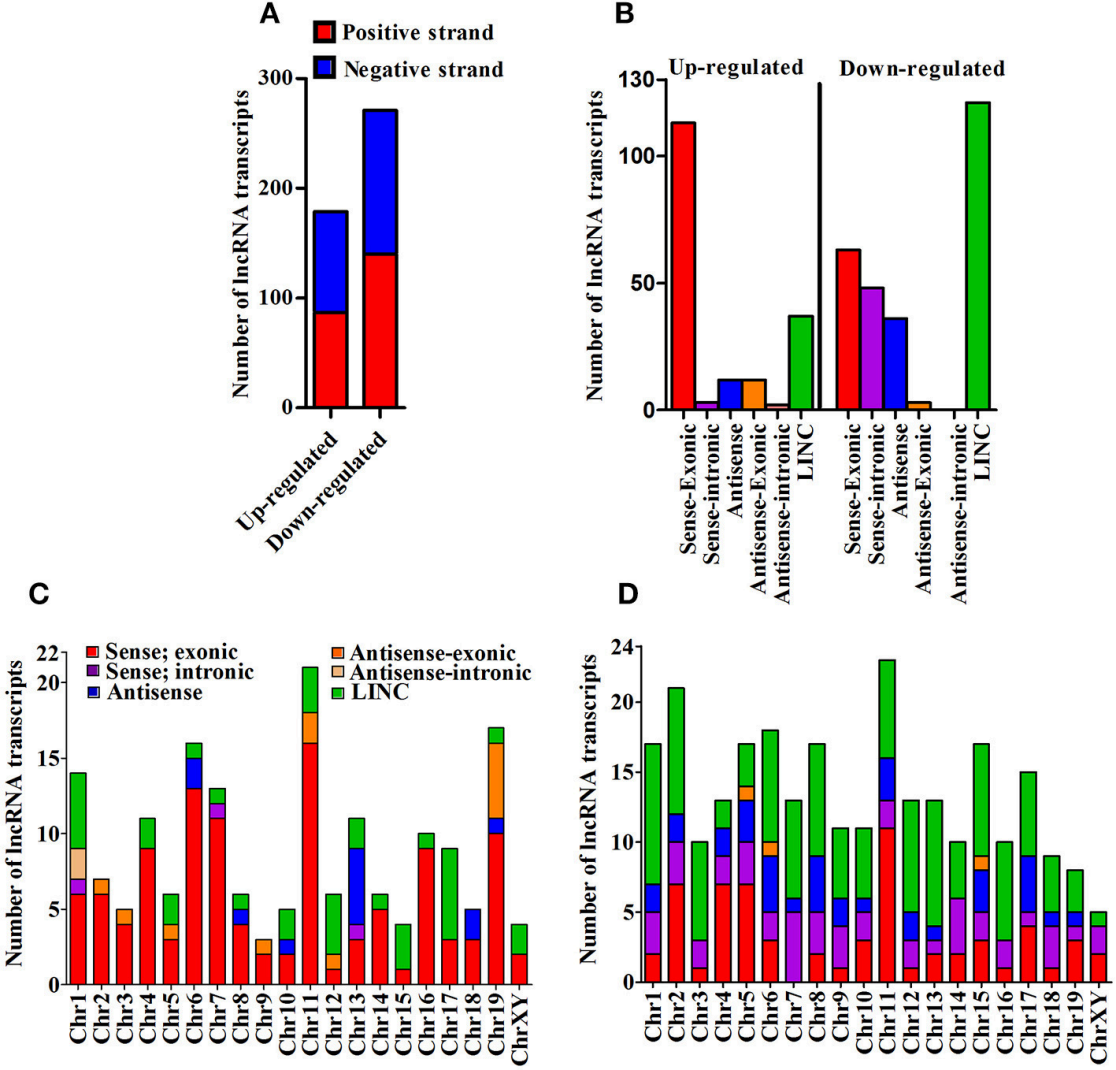
Cataloging of differentially expressed lncRNA transcripts. **(A)** Strand specific origin of differentially expressed lncRNA transcripts; **(B)** Cataloging of lncRNA transcripts based on their origin; **(C)** Chromosomewise distribution of category of up-regulated lncRNA transcripts; and **(D)** Chromosomewise distribution of category of down-regulated lncRNA transcripts.

We next cataloged differentially expressed lncRNAs based on their strand of origin, classification, chromosomal distribution, number of exons, and length. Of up-regulated lncRNAs, the distribution on sense and antisense strands was determined to be 87 (48.6%) and 92 (51.4%), whereas a total of 140 (51.7%) and 131 (48.3%) down-regulated lncRNAs were found to be transcribed from the sense and anti-sense strands, respectively, (Figure 2A). Based on the specifics of their origin, we categorized them into different classes, namely sense-exonic, sense intronic, antisense, antisense-exonic, antisense intronic and LINC (Supplementary Figure 3A). Of 179 up-regulated lncRNA transcripts, a majority (113 or 63.1%) were sense-exonic and the remaining included 37 (20.7%) LINC, 12 antisense-exonic (6.7%), 12 antisense (6.7%), 3 sense-intronic (1.7%), and another 2 antisense-intronic (1.1%). The down-regulated lncRNA transcripts were represented by 121 LINC (44.6%), 63 sense-exonic (23.2%), 48 antisense (17.7%), 36 sense-intronic (13.3%), and 3 antisense-exonic (1.1%) (Figure 2B). Further analysis suggested that most of the differentially expressed lncRNA transcripts are transcribed from chromosome 1, 4, 6, 7, 11, and 13. Majority of the up-regulated lncRNA transcripts are sense-exonic in nature and mainly transcribed from chromosome 4, 6, 7, 11, 16, and 19. However, majority of the down-regulated transcript are LINC in nature and predominantly transcribed from chromosome 1, 2, 6, 7, 15, and 16 (Figures 2C,D). Based on exonic composition, about 27 (15.08%) of up-regulated lncRNA transcripts are mono-exonic, 54 (30.17%) are bi-exonic, and remaining 98 (54.75%) transcripts are multi-exonic. On the otherhand, about 31 (11.44%), 59 (21.77%), and 181 (66.79%) down-regulated transcripts are mono-exonic, bi-exonic and multi-exonic, respectively, (Supplementary Figure 3B). Next, we performed size based cataloging of differentially regulated lncRNAs. Majority of the regulated lncRNA transcripts range from 501 to 2,000 nucleotides (*n* = 106, 59.22% for up-regulated and *n* = 148, 54.61% for down-regulated), followed by those ranging from 200 to 500 nucleotides including both up-regulated (*n* = 37, 20.67%) and down-regulated (*n* = 59, 21.77%) transcripts. About 28 (15.64%) and 46 (16.97%) up- and down-regulated transcripts are within the range of 2,001–5,000 nucleotides. As expected, only a low number of up-regulated (*n* = 8, 4.47%) and down-regulated (*n* = 18, 6.64%) transcripts belong to the category of ≥5,001 nucleotides (Supplementary Figure 3C).

**Figure 3:**
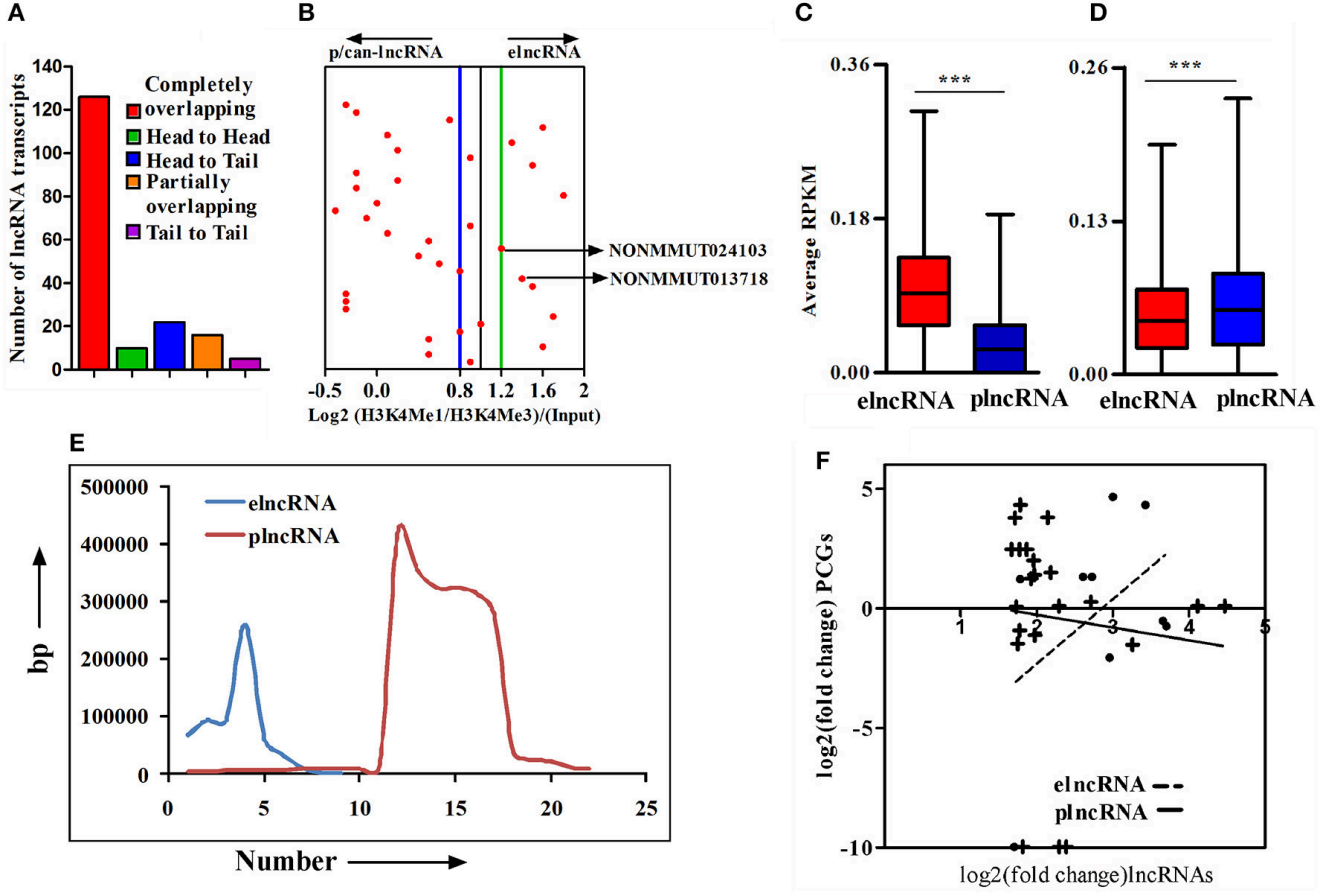
Cataloging of up-regulated lncRNA transcripts based on chromatin signatures. **(A)** cataloging of up-regulated lncRNA transcripts based on their orientation with respect to nearby PCGs; **(B)** Representative plot showing log2 ratio of chromatin signatures H3K4Me1 and H3K4Me3 (after normalization with input) around 4 window of transcription start site (TSS) of lncRNAs; **(C)** Average normalized RPKM (reads per kilobase million) values of H3K4Me1 in elncRNAs and plncRNAs; **(D)** Average normalized RPKM (reads per kilobase million) values of H3K4Me3 in elncRNAs and plncRNAs; **(E)** Distribution of distances of elncRNAs and plncRNAs from their respective nearby genes; and **(F)** Correlation between fold changes of elncRNAs and plncRNAs and their closest protein coding genes (PCGs), respectively. The linear regression curves of the best fit are shown as dotted line for elncRNA and solid line for plncRNA. ***P ≤ 0.001.

An important chromatin signature for identification of enhancers is the combination of H3K4Me1 and p300 binding in the absence of H3K4Me3, a mark that has been classically associated with active or poised TSSs. Accordingly, the ratio of H3K4me1/H3K4me3 around TSSs is a useful indicator to segregate lncRNAs into enhancer-associated (elnc) or promoter-associated (plnc) RNAs. We, therefore, recorded the TSSs of up-regulated lncRNAs and their nearest PCGs from the NONCODE database and UCSC genome browser, respectively. The combinatorial evidence of TSS, chromosomal origin, and relative position of lncRNAs to PCGs was then utilized to classify up-regulated lncRNAs into completely overlapping, partially overlapping, head to head, head to tail, and tail to tail category (Supplementary Figure 4A). A majority of up-regulated lncRNAs (126 out of 179) were determined to be completely overlapping and a few (*n* = 16, 8.92%) were from loci partially overlapping with PCGs. Of those remaining, 10 (5.92%), 22 (12.29%), and 5 (2.79%) transcripts were belonging to head to head, head to tail, and tail to tail orientation, respectively, (Figure 3A). Based on all the cataloging, we excluded lncRNA transcripts belong to classes sense-exonic, antisense-exonic and sense-intronic lncRNAs, to prevent the confounding influence of reads of chromatin and epigenetic signatures from the overlapping mRNAs. Furthermore, orientation of lncRNAs with respect to nearest PCGs also eliminated any ambiguous LINC lncRNAs, for which the TSSs are located within 2 kb region of both the head and tail ends of nearby PCGs. We, however, retained antisense lncRNAs for which the location of TSSs was 2 kb beyond either end of nearby PCGs. We next employed ChIP-seq data for enriched chromatin state around a 4 kb window of the TSS to determine the Log2(H3K4me1: H3K4me3) ratio for classifying the up-regulated lncRNAs as elncRNA (ratio of ≥1.2) or plnc/can-lncRNA (ratio of ≤ 0.8). Such quantitative analysis of H3K4Me1/H3K4Me3 ratio allowed for the designation of a total of 9 and 22 transcripts, respectively, as elnc and plncRNAs, whereas 4 transcripts did not clearly qualify for either category (Figure 3B). Genomic annotations of elncRNAs, plncRNAs and their respective nearby PCGs are presented in Supplementary Table 2.

We further compared average normalized reads for a 4 kb window around the TSSs of elnc and plncRNAs to ensure that elncRNAs and plncRNAs are enriched with H3K4Me1 and H3K4Me3, respectively. As expected, normalized average read densities for H3K4Me1 in elncRNA were significantly higher than plncRNA, while those for H3K4Me3 were significantly higher for plncRNA (Figures 3C,D). Normalized RPKM values against the positions of 4 kb window are presented in the Supplementary Figures 4B,C. Because mechanistic investigations of the regulation of PCGs have implicated elncRNA interactions with looping factors to facilitate chromosomal looping between the enhancer and the promoter(s) of target gene(s), we captured the distances of up-regulated elncRNAs and plncRNAs in relation to the position of proximal PCGs. This analysis suggested that on an average, elncRNAs were located in close proximity to the PCGs, when compared to plncRNAs (Figure 3E). Moreover, to test whether transcription of elncRNAs is responsible for the regulation of nearby PCGs, we estimated the correlation between changes in the expression of elncRNAs (*r*^2^ = 0.3) and plncRNAs (*r*^2^ = 0.01) with their nearby PCGs. As shown, correlation of expression of elncRNAs and their nearby PCGs was determined to be stronger than plncRNAs (Figure 3F).

**Figure 4:**
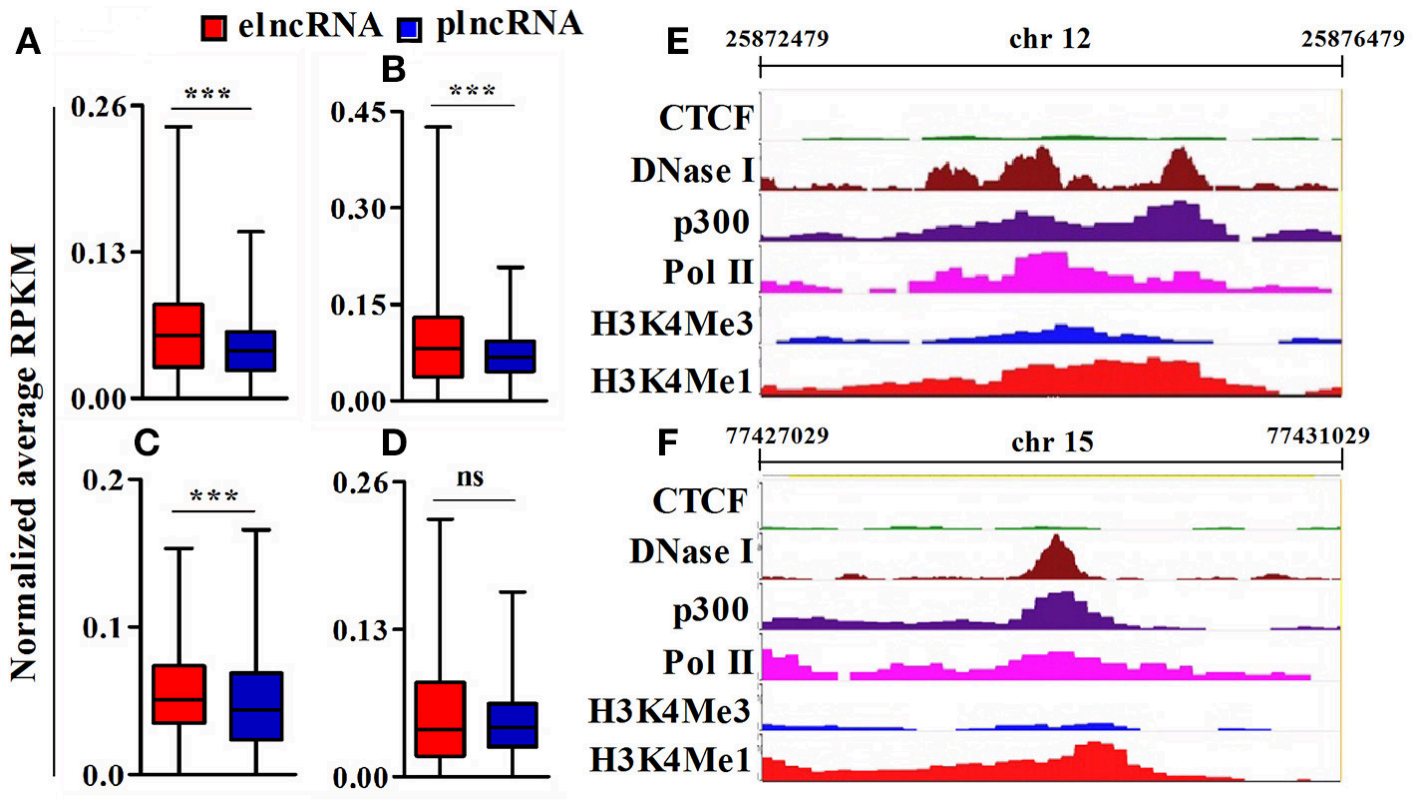
Analysis of epigenomic signatures around transcription start site (TSS) of elncRNAs and plncRNAs. **(A–D)** Average normalized RPKM (reads per kilobase million) values of RNA polII, p300, DNaseI hypersensitivity site, and CCCTC binding factor (CTCF) in elncRNAs and plncRNAs, respectively; **(E,F)** Contrast chromatin (H3K4Me1 and H3K4Me3) and epigenetic (RNA polII, p300, DNaseI hypersensitivity and CTCF binding site) landscapes (in mouse lungs) around TSS of NONMMUT013718 and NONMMUT024103 elncRNAs, respectively. ***P ≤ 0.001 and ns = non-significant.

To identify active elncRNAs, we further subjected our dataset for the analysis of other epigenetic signatures, namely RNA pol II, p300, DNaseI hypersensitivity site, and CTCF, within the same 4 kb window around the TSSs of elnc and plncRNAs. The normalized average RPKM values suggested significantly higher read densities of RNA pol II, p300, and DNaseI hypersensitivity sites for elncRNAs in comparison to plncRNAs (Figures 4A–C), but not for CTCF (Figure 4D). Normalized RPKM values against the positions of 4 kb window are presented in the Supplementary Figures 5A–D. Based on epigenetic landscapes, we identified 3 active elncRNA transcripts NONMMUT013718 (Figure 4E), NONMMUT024103 (Figure 4F), and NONMMUT013717 (a splice variant of NONMMUT013718).

**Figure 5:**
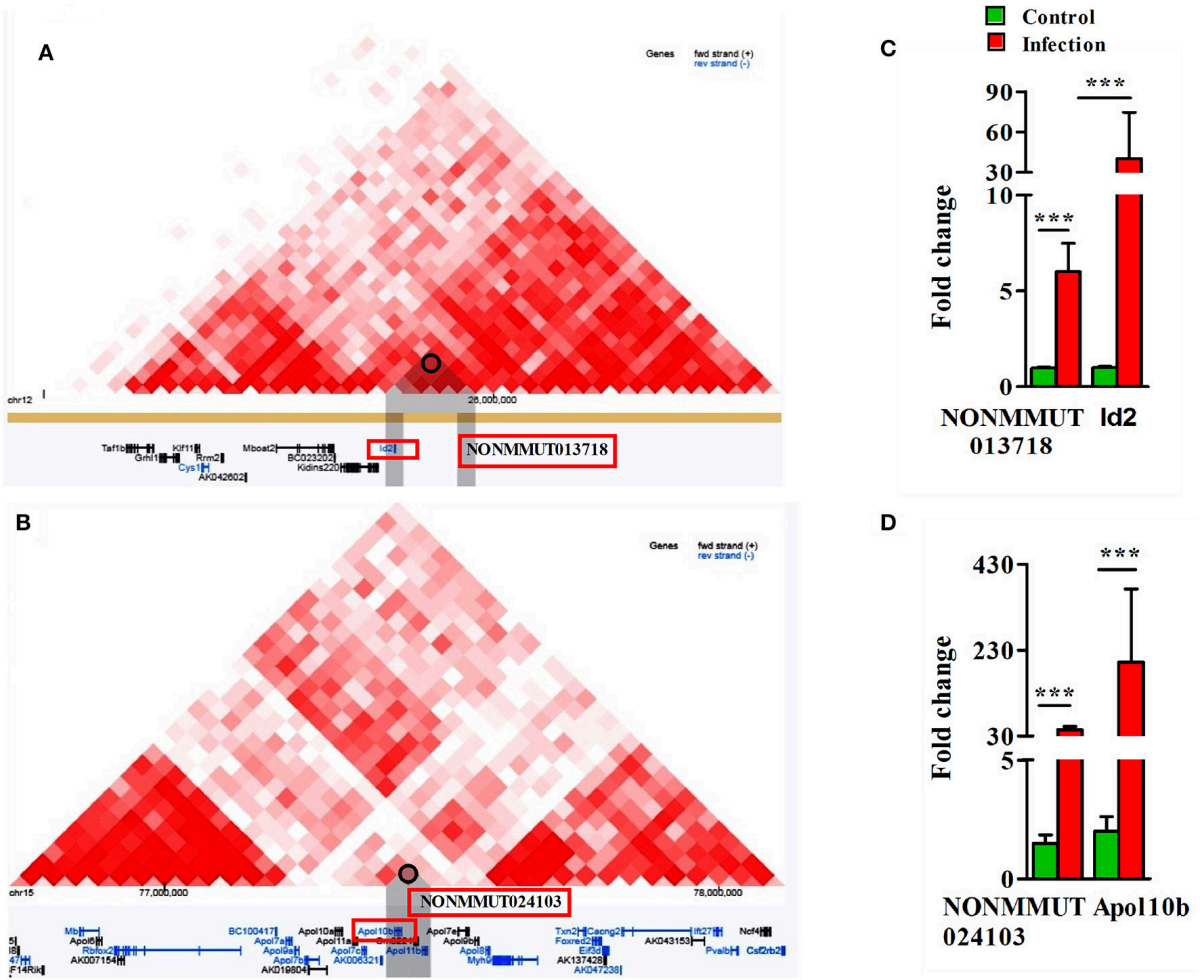
Analysis of Hi-C interaction and qPCR. **(A)** The triangle heatmap of Hi-C tracks of NONMMUT013718 elncRNA and its proximal PCG ID2 and their interacting loci; **(B)** The triangle heatmap of Hi-C tracks of NONMMUT024103 elncRNA and its proximal PCG Apol10b and their interacting loci; **(C,D)** Expression of NONMMUT013718 and NONMMUT024103 elncRNA and their respective target genes ID2 and ApoL10b in infected mouse lungs (*n* = 4). The error bars represents standard error of mean (SEM) and the levels of significance are shown as ***P ≤ 0.001.

To delineate the possibility of interactions between elncRNAs and the promoters of their nearby PCGs, we sequentially analyzed both Hi-C and virtual 4C profiles by constructing a window around the genomic coordinates of active elncRNAs by 3D Epigenome browser. The triangular heatmap for elncRNA NONMMUT013718 demonstrates the potential for interactions with the PCG ID2 (Figure 5A). To further validate this observation and to identify elncRNA regions in contact with the promoter of Id2, we applied virtual 4C profiles supported by 3D genome browser. There are several points of contact for the promoter of Id2 within a 1 Mb window around the anchoring point, including the highest peak representing the potential for interactions with NONMMUT013718 elncRNA (Supplementary Figure 6A). On the other hand, NONMMUT024103 elncRNA regulatory region is just 2.4 kb upstream and relatively adjacent to the promoter of nearby Apol10b gene and a signal of interaction between these two loci is evident in Hi-C heat map (Figure 5B). Similarly, virtual 4C profile analysis also indicates that the promoter of Apol10b lies in apparent contact with NONMMUT024103 elncRNA (Supplementary Figure 6B). Finally, to ascertain whether or not higher expression of elncRNAs NONMMUT013718 and NONMMUT024103 correlates with the expression of their respective target genes ID2 and Apol10b, we performed qRT-PCR on the RNA from lungs of mice infected with *R. conorii*. We observed higher expression of both elncRNAs NONMMUT013718 and NONMMUT024103 as well as their target genes ID2 and Apol10b in the mouse lungs in response to infection (Figures 5C,D).

**Figure 6:**
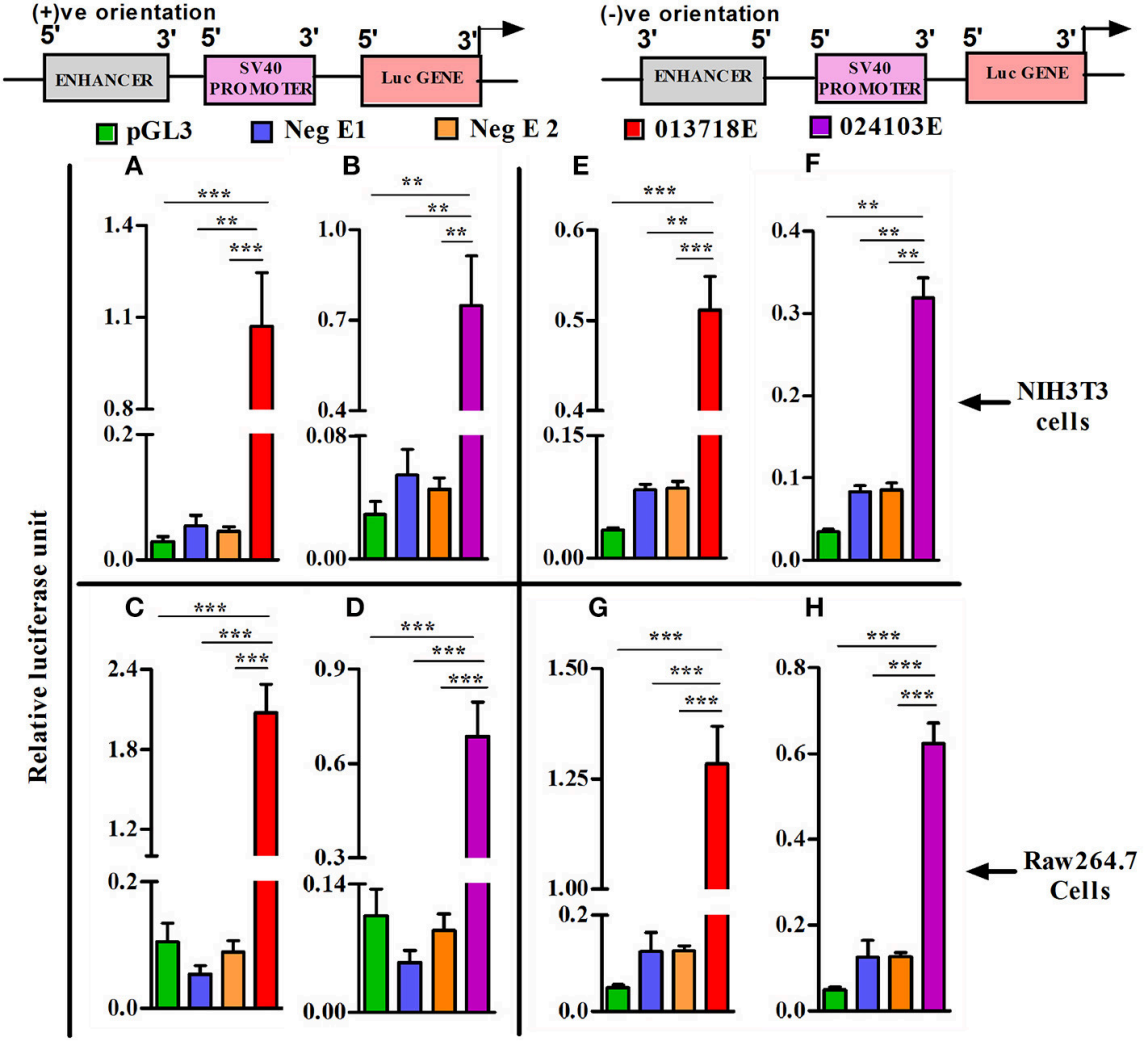
Heterologous reporter assays demonstrating enhancer activity of elncRNA loci. Co-transfection of enhancer reporter construct (sense or antisense orientation) and Renilla reporter plasmid in NIH3T3 cells and Raw 264.7 murine macrophages. **(A,B)** Transfection of NONMMUT013718 and NONMMUT024103 elncRNA constructs (sense orientation) in NIH3T3 cells, and **(C,D)** in Raw 264.7 murine macrophages, respectively; **(E,F)** Transfection of NONMMUT013718 and NONMMUT024103 elncRNA constructs (anti-sense orientation) in NIH3T3 cells, and **(G,H)** in Raw 264.7 murine macrophages, respectively. The data are presented as Mean ± SEM for six independent (*n* = 6) experiments. The negative controls are blank promoter plasmid or inserts with minimal H3K4Me1/H3K4Me3 ratio (Neg E1 and E2). The levels of significance are shown as **P ≤ 0.01; ***P ≤ 0.001.

To assess the potential regulatory effects of NONMMUT013718 and NONMMUT024103 elncRNAs on nearby PCGs, we performed dual luciferase assay using NIH3T3 and RAW264.7 cells in light of their high transfection efficiency. For NONMMUT013718 elncRNA, we observed a significant increase of luciferase activity, suggesting its ability to drive the downstream PCG in comparison to the blank plasmid (pGL3) as well as negative controls in both NIH3T3 and RAW264.7 cells. Similarly, increased luciferase signal activity was also evident in case of NONMMUT024103 elncRNA, when compared with blank pGL3 and corresponding negative controls (Figures 6A–D). To further investigate whether transcriptional activation of nearby PCGs by elncRNAs is orientation independent, we cloned the genomic regions of both elncRNAs in reverse orientation in pGL3 promoter plasmid. Consistent with the findings above, both elncRNAs (NONMMUT013718 and NONMMUT024103) in the reverse orientation significantly enhanced luciferase signal in comparison to the blank plasmid and negative controls (Figures 6E–H).

**Figure 7:**
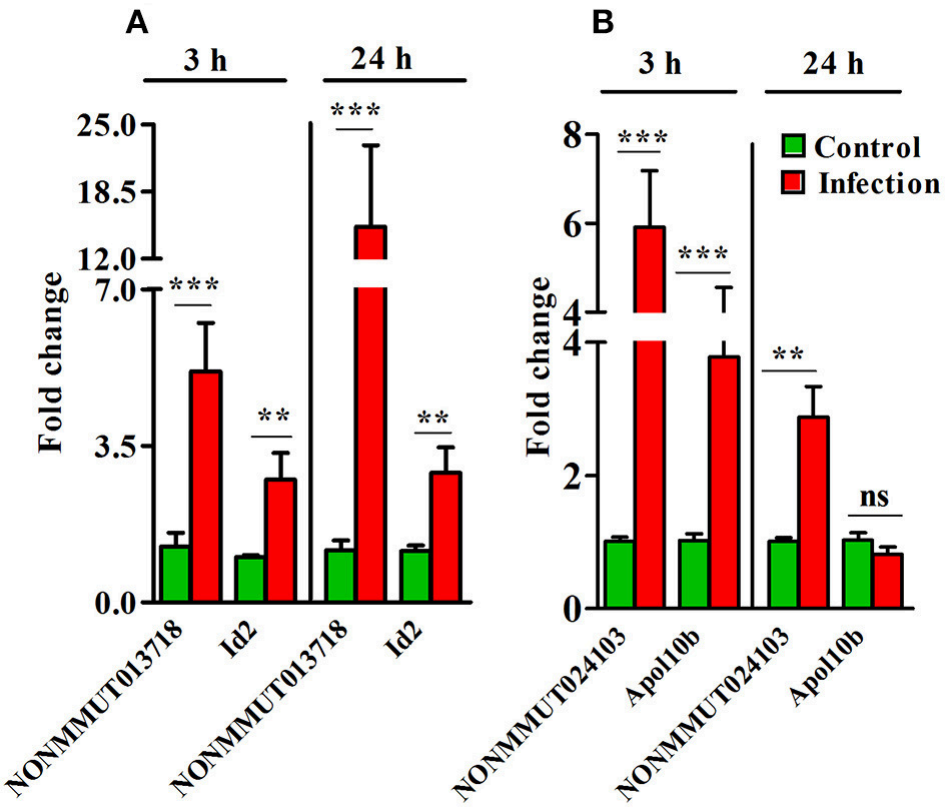
Expression of elncRNAs and their targets in an *in vitro* model of *R. conorii* infection. **(A)** Expression of NONMMUT013718 and its target Id2 in murine Raw264.7 macrophages after infection with *R. conorii* for 3 and 24 h (*n* = 6); **(B)** Expression of NONMMUT024103 and its target Apol10b in murine SVEC endothelial cells after infection with *R. conorii* for 3 and 24 h (*n* = 4). The error bars represent standard error of mean (SEM) and the levels of statistical significance are shown as **P ≤ 0.01; ***P ≤ 0.001; and ns, non-significant.

Importantly, pulmonary vascular cells (endothelial cells from different vascular structures, smooth muscle cells, and adventitial fibroblasts) comprise one of the main functional and structural cell types of the lung and resident macrophages are located in close proximity to the epithelial surface of the respiratory system. Since endothelial cells and macrophages are the predominant targets of rickettsial infection, we carried out q-RT-PCR measurements on RNA isolated from murine RAW264.7 macrophages and endothelial cells (SVECs) infected with *R. conorii* for 3 and 24 h. The expression of both elncRNA NONMMUT013718 and its target ID2 in infected macrophages was significantly higher than mock-infected controls at both 3 and 24 h post-infection (Figure 7A), while that of NONMMUT024103 elncRNA and its target Apol10b both were below the range of detection at either 3 or 24 h post- infection (data not shown). On the other hand, expression of elncRNA NONMMUT024103 and its target Apol10b was significantly higher in SVECs at 3 h post-infection, while only modest increase in NONMMUT024103 was noticeable at 24 h (Figure 7B). Expression of NONMMUT013718 elncRNA remained below the limit of detection, whereas its target ID2 remained below the level of control at both 3 and 24 h of infection in SVECs (Supplementary Figure 7).

**Figure 8:**
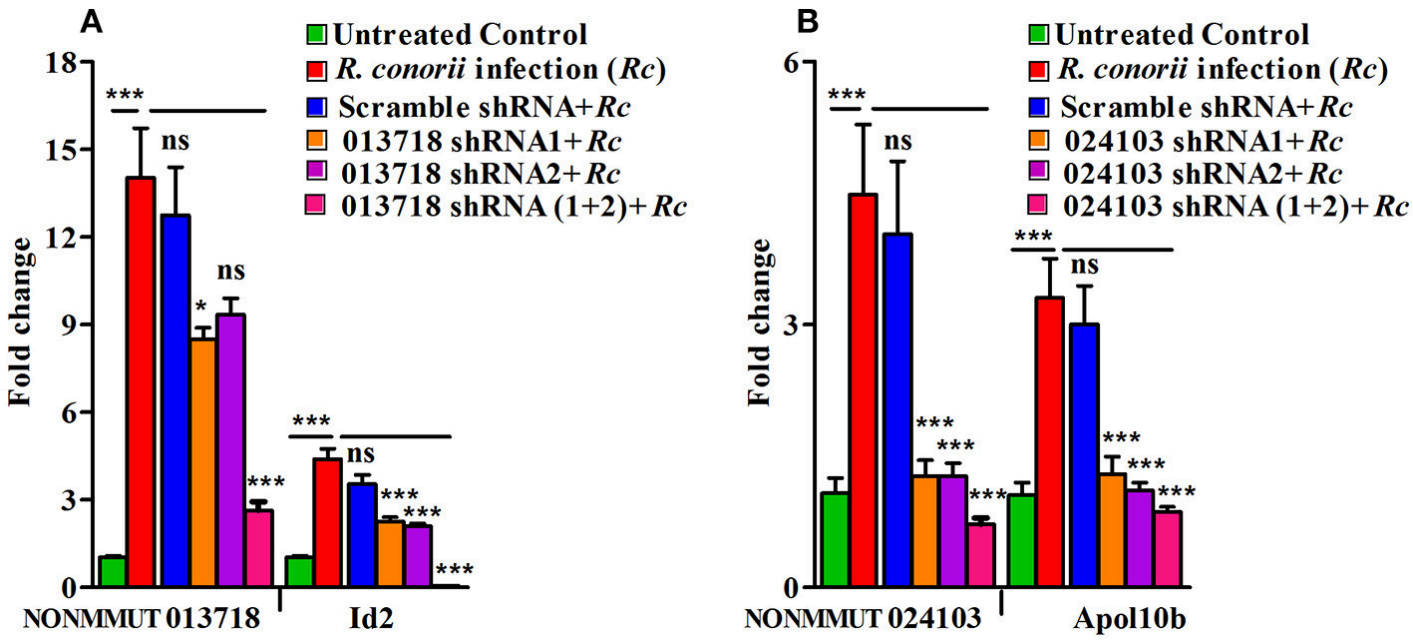
shRNA mediated knock-down of elncRNAs and its target genes during rickettsial infection. Endogenous mRNA expression of Id2 and Apol10b in RAW264.7 macrophages and SVEC endothelial cells after trasfection of NONMMUT013718 shRNA and NONMMUT024103 shRNAs, respectively, following infection with *R. conorii* for 24 and 3 h, respectively. pLKO.1 scramble plasmid was used as a negative control. Knock-down efficiency of **(A)** NONMMUT013718 and its target gene Id2; and **(B)** NONMMUT024103 and its target gene Apol10b were quantified by qPCR. The error bars represented standard error of mean (SEM) and the levels of statistical significance are shown as *P ≤ 0.05; ***P ≤ 0.001; and ns, non-significant.

To further confirm the functional role of NONMMUT013718 and NONMMUT024103 in activation of their respective target genes ID2 and Apol10b, we constructed shRNA plasmids against the target site of both NONMMUT013718 and NONMMUT024103 elncRNAs. We chose RAW264.7 macrophages and SVECs for shRNA mediated knock-down based upon higher expression of NONMMUT013718 and NONMMUT024103 elncRNAs, respectively. We verified transfection and knock-down efficiency of NONMMUT013718 and NONMMUT024103 elncRNAs in RNA isolated from RAW264.7 macrophages and SVECs infected with *R. conorii* for 3 and 24 h, respectively. After infection following transfection, expression level of NONMMUT013718 and NONMMUT024103 was found to be significantly lower. Remarkably, knock-down of NONMMUT013718 and NONMMUT024103 down-regulated the expression of ID2 gene in macrophages, and Apol10b in SVECs (Figure 8).

## Discussion

One of the most remarkable findings of the Human Genome Project is that only about 2% of the DNA accounts for ~20,000 protein-coding genes. Accordingly, it has become increasingly apparent within the past few years that noncoding genome plays an important role in the regulation of coding genome and substantial progress has been made in assessing the contributions of small, single-stranded noncoding microRNAs as regulatory determinants of host responses following infection, immunization, and autoimmunity (39, 40). lncRNAs have been estimated to constitute about 70–90% of the genomic dark matter, which is generically defined as the transcribed yet untranslated component of the human genome. Although loss-of-function approaches have implicated lncRNAs in the biology of both innate and adaptive immune cells during inflammatory insults, their involvement in host responses and pathogenesis of intracellular bacteria remains to be explored to enhance our understanding of their roles in microbial infections. In the present study, we have investigated the global lncRNA profile in the lungs of susceptible murine hosts during infection with *R. conorii*, the causative agent of Mediterranean spotted fever. For this first investigation, we employed a well-established mouse model based on its documented versatility to understand the pathogenesis of human spotted fever group rickettsioses and focused on the lungs as one of the major organ systems targeted by rickettsiae during *in vivo* infection (1, 41). Surprisingly, our RNA sequencing analysis revealed a relatively large number of differentially expressed non-coding transcripts in the lungs during *R. conorii* infection, including 179 up-regulated and 271 down-regulated transcripts of >200 bases length annotated as the lncRNAs in the NONCODE (v4.0) database, an integrated web-based resource dedicated to the analysis of non-coding RNAs (excluding tRNAs and rRNAs). To ensure the consistency of observations, we further validated increased expression of four randomly selected lncRNAs via an independent quantitative PCR-based approach. Thus, although our global analysis was suggestive of potentially important roles for lncRNAs in the determination of coding transcriptome of the host cells or organ systems following infection, an obvious next step emerging from the initial studies was to identify lncRNA candidates with either known functions or potential functional implications. To this end, in-depth analysis of the patterns of histone protein methylation from ChIP sequencing data in conjunction with high throughput chromosome conformation capture sequencing and visualization of Hi-C data in a virtual 4C format revealed that two elncRNAs NONMMUT013718 and NONMMUT024103 may have downstream roles in the regulation of their respective target genes Id2 and Apol10b during rickettsial infection.

Histone proteins in the eukaryotic genome undergo several covalent post-translational modifications, including acetylation, methylation, phosphorylation, ubiquitylation, and sumoylation. Such modifications have a profound effect on gene expression by altering chromatin structure or recruiting modifiers of chromatin activity. Single and trimethylation of histone protein 3 at lysine 4 (H3K4Me1 and H3K4Me3) are well-established features for cataloging of enhancer and promoter elements in the genome (18, 29). Specifically, active enhancers are highly enriched with the presence of H3K4me1 and p300/CBP transcription co-activator binding sites. p300/CBP are two similar acetyltransferases in humans, which play a central role in the pathways responding to intracellular, extracellular, and intercellular signals. These pathways control key cellular functions via altering expression of target genes, through the action of p300/CBP in the nucleus. Open chromatin in the genome is generally ascertained by DNaseI hypersensitivity mapping, however other regulatory elements of the genome such as the promoters, silencers, and insulators also possess DNaseI hypersensitivity sites, rendering exclusive analysis of these sites insufficient for the identification of enhancers (42). Therefore, DNaseI hypersensitivity sites in conjuntion with the p300/CBP protein in the genomic regions indicate the presence of enhancer elements in the genome. p300/CBP recruits RNA polymerase II at the site of enhancers for transcription of elncRNAs (43) and a previous study has documented that a number of uni- and bidirectional elncRNA transcripts have higher occupancy of H3K4Me1, p300/CBP, and RNA pol II (16, 43). On the other hand, CCCTC-binding protein (CTCF) in the genome is considered a hallmark for potential insulator elements that inhibit transcription. Presence of CTCF binding sites in the same domain of enhancer and promoter of the PCG blocks the interaction between these regulatory elements of the genome (31). Based on the H3K4Me1 to H3K4Me3 ratio, we stringently annotated up-regulated lncRNA transcripts into elncRNAs and plncRNAs. Our analysis showed that most of the elncRNA candidates are from intergenic regions and tend to be closely located to the nearby protein coding genes. Moreover, a pattern of positive correlation of expression between elncRNAs and nearby PCGs indicates that these elncRNAs might be associated with nearby PCGs for their expression and function. In addition, examination of the transcription start sites of NONMMUT013718 and NONMMUT024103 within the Gene Expression Omnibus dataset on mouse lung tissues further suggests the likelihood of active elncRNA functions for these transcripts. Enhancers have been proposed to interact with their target promoters by different mechanisms based on their genomic positions. For example, enhancers interact with the PCGs either by transcription of elncRNAs from distal regulatory loci or by formation of chromatin loops with nearby genes (44, 45). Our findings of the ability of NONMMUT013718 and NONMMUT024103 to drive the expression of downstream luciferase reporter genes in an orientation-independent manner and the effects of shRNA-mediated elncRNA knockdown on the expression of target genes during infection suggest the possibility of elncRNA interactions with the promoter of PCGs Id2 and Apol10b, respectively. Intriguingly, both of these elncRNAs and their putative targets are found to be highly up-regulated during *R. conorii* infection, which lends further support to the plausible involvement of their potential regulatory roles in the activation of respective proximal PCGs.

*In vitro* models of infection to delineate interactions between rickettsiae and their target host cells have long been established and routinely used in light of better tractability and direct applicability to decipher the fine details of cellular, molecular, and pathophysiological mechanisms of disease pathogenesis. Although pathogenic rickettisae as intracellular parasites display a predilection to primarily target microvascular endothelial cells lining the small and medium-sized vessels, invasion and infection of macrophages at the site of arthropod feeding during natural transmission to the mammalian hosts and in established needle inoculation-based animal models mimicking human disease is also evident (46). A recent study further documents notable differences in the ability of virulent vis-à-vis avirulent strains of rickettsiae to proliferate in macrophage-like cells *in vitro* as an important determinant of pathogenicity (47). In this context, an intriguing finding of the present study is the up-regulation of elncRNA NONMMUT013718 and Id2 during *R. conorii* infection of RAW264.7 macrophages, whereas only endothelial cells exhibit induced expression of NONMMUT024103 and Apol10b in response to infection. In light of previous evidence indicating that a significant fraction of lncRNAs show lineage-specific expression (48), we interpret these results as the host cell-specific and selective response in regards to the expression of a particular elncRNA.

The proteins belonging to the apolipoprotein (Apol) family are highly conserved across species and are generally thought to be involved in lipid transport and metabolism, due mainly to the association of Apol-1 as a subclass of high-density lipoproteins in human blood. Amongst Apols, the Apol1 in humans is unique in that it can be secreted due likely to its N-terminal signal peptide, accounts for the trypanosome lytic factor activity of human serum, and displays structural and functional similarities with Bcl-2 proteins involved in the regulation of apoptosis and autophagy. In addition, cultured human umbilical vein endothelial cells express CG12_1 (Apol-like) gene as a delayed early marker of inflammation in response to *in vitro* treatment with tumor necrosis factor-α and CG12_1 has been demonstrated to be specifically expressed in endothelial cells lining the normal and atherosclerotic iliac artery and aorta (49). The functions of other members of the Apol family classified on the basis of sequence homology to Apol1, however, are not well-understood. In a recent study, mouse Apol9a and Apol9b have been documented as bonafide interferon-stimulated genes (ISGs) with antiviral activity against Theiler's murine encephalomyelitis virus (50). Our laboratory has previously reported on the ISG response to *R. conorii* infection and its role in the interference with bacterial replication in human microvascular endothelial cells (51), suggesting the possibility of a potential link between higher expression of NONMMUT024103 elncRNA and Apol10b and the type 1 interferon response of host cells.

Inhibitor of DNA binding (Id) proteins, including Id1, Id2, Id3, and Id4, are basic helix-loop-helix (bHLH) transcription regulators. Although other bHLH proteins are known to regulate the transcription of a number of target genes by functioning as homo- or heterodimers, interactions between ubiquitously expressed E protein transcription factors and Id proteins by virtue of their destitute DNA-binding domain inhibit the formation of transcriptionally active complexes (52). Consequently, Id proteins are involved in the control of multiple cellular processes, including differentiation, proliferation, and fate determination (53, 54). Id2 also performs multiple essential functions in the hematopoietic system for the development of dendritic cells, NK cells, intraepithelial T cells, and lymphoid tissue inducer cells (55–57). Although the findings of this study are the first to demonstrate increased expression of NONMMUT0013718 and Id2 in macrophages but not endothelial cells infected *in vitro*, it remains to be determined whether changes in the lungs during *in vivo Rickettsia* infection are due to increased transcription within target host cells, increased recruitment of inflammatory cells, or possibly a combinatorial effect of both. During *Listeria monocytogenes* infection, Id2 regulates gene expression by CD8^+^ T cells and determines the magnitude of effector responses, suggesting a mechanism involving Id2 governed and E protein-mediated survival and differentiation of mature T cells (58). Published findings from the mouse model of rickettsiosis employed in this study yield evidence for increased expression of T cell targeting chemokines CXCL9 and CXCL10 in the lungs and infiltration of CD8+ T cells in the perivascular space around *Rickettsia*-infected microvessels (59). In addition, CD8+ T cells have been implicated in protective immunity against rickettsial infections, which is mediated in part by the cytotoxicity toward infected cells. Therefore, further studies to investigate potential regulatory roles of NONMMUT0013718 and Id2 in the determination of host immune responses to rickettsiae with particular attention to T cell mediated immunity are justified and currently ongoing.

## Conclusion

In conclusion, the present study reports on differential expression of a number of lncRNA transcripts in the lungs as one of the prominent target organs in an established mouse model of rickettsial infection. From this subset of lncRNAs, we have identified two active elncRNAs through systematic application of genomics, epigenomics, and functional analysis to further demonstrate selective, cell-specific regulation of these lncRNAs and their potential target genes in vascular endothelial cells and macrophages as the target host cells *in vitro*. Given the data suggesting contributions of lncRNA-based regulatory networks in the modulation of host gene expression and differentiation as well as homing of T cells, further in-depth mechanistic enquiries of these versatile biological mediators in host-pathogen crosstalk and pathogenesis should unveil new strategies to counteract bacterial infections.

## Author Contributions

IC designed and performed all *in vitro* experiments. HN and AS performed animal experiments and provided the samples for RNA-seq. IC and KK performed all the bioinformatics analysis. HN and YF assisted in the analysis of RNA sequencing data. IC and SS supervised the study. IC and SS wrote and edited the manuscript and acquired funding to support this work. HN, AS, KK, and YF helped in editing and reviewing the manuscript. SS provided the resources for completion of this study. All authors read and approved the final manuscript.

### Conflict of Interest Statement

The authors declare that the research was conducted in the absence of any commercial or financial relationships that could be construed as a potential conflict of interest.

## Supplementary Material

Supplementary Table 1 Total reads from RNA sequencing experiments and the number of reads mapping to coding (mRNA) and non-coding (ncRNA) transcripts.

Supplementary Table 2 Genomic annotation of up-regulated elncRNAs, plncRNAs and the nearby PCGs.

Supplementary Table 3 Primer sequences for elncRNA and mRNA expression, and cloning.

Supplementary File 1 The shRNA target sites are highlighted with colors.

Supplementary Figure 1 Schematic representation of RNA-sequencing and downstream analysis.

Supplementary Figure 2 Volcano plot displaying differentially expressed lncRNA transcripts (cut off value 3-fold, *P*-value -Log_10_ ≤ 0.05 ~ 1.3). Red and green dots on the x-axis represent positively and negatively regulated transcripts, respectively. Values on the y-axis represent the level of significance.

Supplementary Figure 3 **(A)** Strategies applied for cataloging of differentially expressed lncRNA transcripts based on their origin; **(B)** Cataloging of lncRNA transcripts based on their exon numbers; and **(C)** Cataloging of lncRNA transcripts based on their lengths.

Supplementary Figure 4 **(A)** Strategies applied to identify orientation of up-regulated lncRNA transcripts with their nearby protein coding genes (PCGs); **(B)** Histogram depicting density of average normalized RPKM (reads per kilobase million) values of H3K4Me1 in elncRNAs and plncRNAs; and **(C)** Histogram depicting density of average normalized RPKM (reads per kilobase million) values of H3K4Me3 in elncRNA and plncRNAs around 4 kb window of transcription start site.

Supplementary Figure 5 Histogram depicting density of average normalized RPKM (reads per kilobase million) values of **(A)** RNA PolII; **(B)** p300; **(C)** DNaseI hypersensitivity site; and **(D)** CCCTC binding factor (CTCF) around 4 kb window of transcription start site of elncRNAs and plncRNAs, respectively.

Supplementary Figure 6 Virtual 4C plot of **(A)** NONMMUT013718 and **(B)** NONMMUT024103 elncRNA. The anchoring point (red line) marks the position of **(A)** Id2 and **(B)** Apol10b, respectively. The contact loci are shown with blue line.

Supplementary Figure 7 Expression of elncRNAs and their targets in an *in vitro* model of *R. conorii* infection. Expression of NONMMUT013718 and its target Id2 in SVECs at 3 and 24 h post-infection.

## Abbreviations

SFG: spotted fever group
RMSF: Rocky Mountain spotted fever
MSF: Mediterranean spotted fever
FANTOM: Functional annotation of the mammalian genome
ENCODE: Encyclopedia of DNA Elements
ncRNAs: non-coding RNAs
lnc: long non-coding
elnc: enhancer long non-coding
plnc: promoter long non-coding
PCGs: protein coding genes
TSS: transcription start site
GEO: gene expression omnibus
RIN: RNA integrity number
RKPM: reads per kilobase million
qRT-PCR: Quantitative real-time PCR
LINC: long intergenic non-coding
TES: transcription end site
CTCF: CCCTC binding protein
SVEC: SV40-transformed mouse endothelial cells
shRNA: short hairpin RNA
RNA-seq: RNA sequencing
rRNA: ribosomal RNA
tRNA: transfer RNA
ChIP-Seq: Chromatin Immunoprecipitation sequencing
H3K4Me1: monomethylation of histone H3 at lysine 4
H3K4Me3: Trimethylation of histone H3 at lysine 4
Apol: apolipoprotein
ISGs: interferon-stimulated genes
Id: Inhibitor of DNA binding
bHLH: basic helix-loop-helix.
