# Supplementary material for: Enhancer Associated Long Non-coding RNA Transcription and Gene Regulation in Experimental Models of Rickettsial Infection
Source: Front Immunol. 2019 Jan 9;9:3014. doi: 10.3389/fimmu.2018.03014 (PMC6333757; doi:10.3389/fimmu.2018.03014)
Supplement: Supplementary Table 1 — Total reads from RNA sequencing experiments and the number of reads mapping to coding (mRNA) and non-coding (ncRNA) transcripts. [file Table_1.docx]

|  | **Control group** | |  | ***R. conorii* infected group** | |
| --- | --- | --- | --- | --- | --- |
| **Total reads** | |  |  |  |  |
|  | **Mouse no. 1** | 42,433,442 |  | **Mouse no. 1** | 40,105,218 |
|  | **Mouse no. 2** | 45,331,462 |  | **Mouse no. 2** | 69,529,702 |
|  | **Mouse no. 3** | 64,700,088 |  | **Mouse no. 3** | 50,666,662 |
|  |  |  |  |  |  |
| **Reads mapped to ncRNAs (without removing RefSeq genes)** | | | | |  |
|  | **Mouse no. 1** | 12,286,909 |  | **Mouse no. 1** | 15,898,184 |
|  | **Mouse no. 2** | 13,353,483 |  | **Mouse no. 2** | 24,347,064 |
|  | **Mouse no. 3** | 24,143,273 |  | **Mouse no. 3** | 20,631,561 |
|  |  |  |  |  |  |
| **Reads mapped to RefSeq Genes** | | |  |  |  |
|  | **Mouse no. 1** | 12,458,631 |  | **Mouse no. 1** | 13,159,006 |
|  | **Mouse no. 2** | 13,666,657 |  | **Mouse no. 2** | 22,361,967 |
|  | **Mouse no. 3** | 19,632,462 |  | **Mouse no. 3** | 16,699,450 |
|  |  |  |  |  |  |
| **Reads mapped to ncRNAs (after removing RefSeq genes)** | | | | |  |
|  | **Mouse no. 1** | 6,704,424 |  | **Mouse no. 1** | 10,279,080 |
|  | **Mouse no. 2** | 7,447,288 |  | **Mouse no. 2** | 14,737,840 |
|  | **Mouse no. 3** | 15,557,147 |  | **Mouse no. 3** | 13,641,277 |

**Supplementary Table 1.** Total reads from RNA sequencing experiments and the number of reads mapping to coding (mRNA) and non-coding (ncRNA) transcripts.
