# Supplementary material for: Enhancer Associated Long Non-coding RNA Transcription and Gene Regulation in Experimental Models of Rickettsial Infection
Source: Front Immunol. 2019 Jan 9;9:3014. doi: 10.3389/fimmu.2018.03014 (PMC6333757; doi:10.3389/fimmu.2018.03014)
Supplement: Supplementary Table 3 — Primer sequences for elncRNA and mRNA expression, and cloning. [file Table_3.DOC]

**Supplementary Table 3: Primer** sequences for elncRNA and mRNA expression, and cloning.

| **Name** | **Purpose** | **Sequences** |
| --- | --- | --- |
| **18S rRNA FP** | House keeping gene | GTAACCCGTTGAACCCCATT |
| **18S rRNA RP** |  | CCATCCAATCGGTAGTAGCG |
|  | | |
| **NONMMUT024102FP** | lncRNA | CCCCTGGGCACTAGAGAAAGA |
| **NONMMUT024102RP** |  | CATGTCTCCAGCTGATCATGTTTT |
| **NONMMUT007594FP** | lncRNA | AGCATGAGTAATTAGGCCACAAAAT |
| **NONMMUT007594RP** |  | TCCTTGGGCTTTGATGATGAA |
| **NONMMUT013718FP** | lncRNA | TCTGAGTGCCCCATGAAGCT |
| **NONMMUT013718RP** |  | TTGGAAGGATCCTGAGGTTTTG |
| **NONMMUT019215FP** | lncRNA | ACCCCTTGCTCGTTCTTTGA |
| **NONMMUT019215RP** |  | GGTGGACTGGGACCAAGGA |
| **NONMMUT029515FP** | lncRNA | GCCTGCTTCACTGGTCCAA |
| **NONMMUT029515RP** |  | TGTCCAGGAACTCAGGTCTTTCT |
| **NONMMUT001084FP** | lncRNA | CCGCTCCCTCTCCAACACT |
| **NONMMUT001084RP** |  | CTGCAGGCAGTTCTCAAAAAGA |
| **NONMMUT047494 FP** | lncRNA | TTAACTGCGGAGCCGTCTCT |
| **NONMMUT047494 RP** |  | CCGGAGGCTTAGCACAGAAG |
| **NONMMUT003295 FP** | lncRNA | ACGTCCCAGGAGCCTACAGA |
| **NONMMUT003295 RP** |  | CGAACTTGAAGAACTGGTGACAA |
|  | | |
| **ID2FP** | mRNA | GAAAAACAGCCTGTCGGACCA |
| **ID2RP** |  | CCAGGGCGATCTGCAGGT |
| **APOL10B FP** | mRNA | CCGAGACCTGGCTGATCATC |
| **APOL10B RP** |  | CCCATGGCTCCAGAGATAGC |
|  | | |
| **NONMMUT 013718 Flanking FP** | Cloning of NONMMUT013718 enhancer flanking region | TCACTAGGTACCCCGCCTTCAGACGGTTCCAGGA |
| **NONMMUT013718 Flanking RP** | Cloning of NONMMUT013718 enhancer flanking region | CTCATAACGCGTGTCCTTTCCTGATAG CAA TTA CC |
| **NONMMUT024103 Flanking FP** | Cloning of NONMMUT024103 enhancer flanking region | GCT ACT GGT ACC GCA GGG ATC TAC CAT TTG TTG GGA |
| **NONMMUT024103 Flanking RP** | Cloning of NONMMUT024103 enhancer flanking region | TCC TAT ACG CGT TGG GGA ATC AAA GCC AGA GCT GTT G |
|  | | |
| **NONMMUT013718 Flanking CFP** | Cloning of NONMMUT013718 enhancer flanking region reverse orientation | CTC ATA ACG CGT CCG CCT TCA GAC GGT TCC AGG A |
| **NONMMUT013718 Flanking CRP** | Cloning of NONMMUT013718 enhancer flanking region reverse orientation | TCA CTA GGT ACC GTC CTT TCC TGA TAG CAA TTA CC |
| **mm024103 Flanking CFP** | Cloning of NONMMUT024103 enhancer flanking region reverse orientation | TCC TAT ACG CGT GCA GGG ATC TAC CAT TTG TTG GGA |
| **mm024103 Flanking CRP** | Cloning of NONMMUT024103 enhancer flanking region reverse orientation | GCT ACT GGT ACC TGG GGA ATC AAA GCC AGA GCT GTT G |
|  |  |  |
| **Enhancer Neg1 FP** | Cloning for negative enhancer | ATCATAGGTACCAGTCTAACCTCTTCTTCAGCCTCTCCT |
| **Enhancer Neg1 RP** | Cloning for negative enhancer | AGTGAGACGCGTGAAGCAAGGTGGACTGTGATCTAGACA |
| **Enhancer Neg2 FP** | Cloning for negative enhancer | ATCATCGGTACCTCTTATAATGGCAAATGTGAGTGCGGC TC |
| **Enhancer Neg2 RP** | Cloning for negative enhancer | CGAGAGACGCGTAGTTACTCACTCATTTTGTAAGCAA |
|  |  |  |
| **Rvprimer 3** | Sequencing of enhancer inserts | CTAGCAAAATAGGCTGTCCC |
|  |  |  |
| **SV40-F-XhoI** | Modifying pGL3 basic plasmid into pGL3 promoter plasmid | GCTATTCTCGAGTGCATCTCAATTAGTCAGCA |
| **SV40-RP** |  | ACCAACAGTACCGGAATGCC |
|  |  |  |
| **NONMMUT013718 shRNA 1** | Forward oligo | 5'-CCGGCTCCAGGGAGTAAAGAAATTACTCGAGTAATTTC  TTTACTCCCTGGAGTTTTTG-3' |
|  | Reverse oligo | 5'-AATTCAAAAACTCCAGGGAGTAAAGAAATTACTCGAG  TAATTTCTTTACTCCCTGGAG-3' |
| **NONMMUT013718 shRNA 2** | Forward oligo | 5'-CCGGTGCAGCAAGTCGGGAAGATATCTCGAGATATCTT  CCCGACTTGCTGCATTTTTG-3' |
|  | Reverse oligo | 5'-AATTCAAAAATGCAGCAAGTCGGGAAGATATCTCGAG  ATATCTTCCCGACTTGCTGCA-3' |
|  |  |  |
| **NONMMUT024103 shRNA1** | Forward oligo | 5'-CCGGCGGCCTCAAGAACGGAATTAACTCGAGTTAAT  TCCGTTCTTGAGGCCGTTTTTG-3' |
|  | Reverse oligo | 5'-AATTCAAAAACGGCCTCAAGAACGGAATTAACTCGAGT  TAATTCCGTTCTTGAGGCCG-3' |
| **NONMMUT024103 shRNA2** | Forward oligo | 5'-CCGGACCTAGCTTGAGCCAAGTATACTCGAGTATACTT  GGCTCAAGCTAGGTTTTTTG-3' |
|  | Reverse oligo | 5'-AATTCAAAAAACCTAGCTTGAGCCAAGTATACTCGAGT  ATACTTGGCTCAAGCTAGGT-3' |

**Definition of abbreviations**- shRNA: short hairpin RNA; FP: Forward primer; RP: Reverse primer; CFP: Complementary forward primer; CRP: Complementary reverse primer; Neg: Negative
