# Supplementary material for: Enhancer Associated Long Non-coding RNA Transcription and Gene Regulation in Experimental Models of Rickettsial Infection
Source: Front Immunol. 2019 Jan 9;9:3014. doi: 10.3389/fimmu.2018.03014 (PMC6333757; doi:10.3389/fimmu.2018.03014)
Supplement: Supplementary File 1 — The shRNA target sites are highlighted with colors. [file Data_Sheet_1.doc]

**Supplementary file (The shRNA target sites are highlighted with colors)**

**>NONMMUT013718**

AGCTTCACTGTGTGACGCGCTTTGTGACAAGCTGCCAGGGCCAGGAGCTGTAGCAGCTGCGCTTGAACTTGTGGCTGGCTGCAGTCCCTTGGAAAGGGAAGGGGCACATCTGGAAGGCGGGTCTGAGTGCCCCATGAAGCTCAGGCTGGCTTACCAACCCAAGCAAAGCCTGTAGCAATTTCAAAACCTCAGGATCCTTCCAATTGCCTCAGTGAGCCTTGGAGAAGCCACAGACCTGATACACAAGGCTGAACACGGGGACAGCAGCACTTCCTACTGGCCTCTCATAGGCAGCTGGGGAAACCCCTTCTTCATGCAGCAAGTCGGGAAGATATGGATATCCAACGAGGGGTCTGAAGGTAATGTTGGTCCTGAGCTGATCTCTGTACTTGAGCCTAGAGAAATGTGGAAGCCAGCCTCTGGTTCTGCCCCAGAGAAGCTCTCACATCTGTCCCTCACAGTCTGTGTCTGTGGCAGGTTACTTCCTCTTTGAACCTCAGTTTCCTTATCTGTCAGATGGAGCAAACATGGGGACCTGCCATGGGAGAACCCCAGCTCATGCAGGTAGAAATGTCAGCTCTAAGATCCTGGGCCCCATGCTACCCTCCCTGAGATGCTGCTATGTGAATGTGGAGGGAAAGAAACAGAAGTTACTGCATCTTACTCCATCCTAAGAAAGGCTACAGTGGGCAGCCCTGATTCCCTCCTGCTGTCCAGAACCTGAGAGCCCAGTAAATTGGAGCCTAGGCCACTGTGGCCTTTGAAAGACAGACAGAAGACTTCCAGACACGCAGCAAAGATAAGAAAGCTGCAAGGATGCCAGAGGCCTCCCAACATCCAATCCAGACAGAGCCTTGTCTGAGGTAGAGGTTTCCAATGAGTGAGCCCCAGGAAATCTTAAGTCTTGGGGATATGGCATACTAGCAGAGGGGCAACAATCAGCTCCAGGGAGTAAAGAAATTAAAAGCAGCCCTTGTTGTAGCTTGTGCCAATTTGTATGGTATAATCACTCCTGCCTTGGCTAATGTCATCACTGAAGGCAAATTCAGGACCAGCAAAGCATAGTCAACTGTCCTGAGCAGCAACATCTCACACTCAAAGCATATGAGAACAGATGGCTCTTCCCATAAGATTCTTGCTACCTGCCCAGAGGACCAAGAACAAACAGTGGCATGTCTCGGCTGGAAGACAGAGGAGCAGAAGGGTTGTTGAGTGTTACAGGTTGGCTAGGAAAAGTCCCACACCGGCTTGTGTGTTTGAACACTTGCTCTCCAGCTCATGGTGCTCTCTGGGAAGTTTCTGGAACCTTTTAAAGGTGAGGCTGTGGTGATTTAAAGAATAGTCCCCTTGGGCTCCTATTTTGAATACTTGGTAGAACTGTTTGGGGAGGATTGGGAGGTGTGGCCTTGTTGGAGAAGATGTATCACTGGGGGTGGACTTTGAGGTTCTAAAAGACTCCCAGTGTTCTTCCTCTACCTCGTGGTTGTGGATCAAAATGTGAGCTGATCTTCCCTCCTCCATCACTCATTGTAGTCCTCTGAAACCATAAGCCAAAATAAATGTTTT

**NONMMUT013718 shRNA1**

**NONMMUT013718 shRNA2**

**>NONMMUT024103**

GCTGGAGAAGGTGCCCTGCCACCTAGCTTGAGCCAAGTATAGTTAATCAGCCAAACTCTCTCAACGCCTGGAGAGAGAACCAATCAATCAAGGGAAAGACAGACTTCCAGTAAAAAGCCTGGTCCAGATCAGAAGTTGTTTTCTCTGCTGGAAAGATGTGGATTGAAGTTGTTTCTTTCGGCCTCAAGAACGGAATTAAAGGTGTGACGTCCACGTCGCAGATTTAGACCAGAAACAGATCTTTACTCTTCAGATTTAATAATCATCCCTCCCAGGTTGAAGTGCACAGGCAGCTTGCTTCTGCACTCTTGCAGGTCTCCCTTCCCCCACCCTAGGCCCTCTCC

**NONMMUT024103 shRNA1**

**NONMMUT024103 shRNA2**
