# Supplementary figures and images for: Enhancer Associated Long Non-coding RNA Transcription and Gene Regulation in Experimental Models of Rickettsial Infection
Source: Front Immunol. 2019 Jan 9;9:3014. doi: 10.3389/fimmu.2018.03014 (PMC6333757; doi:10.3389/fimmu.2018.03014)

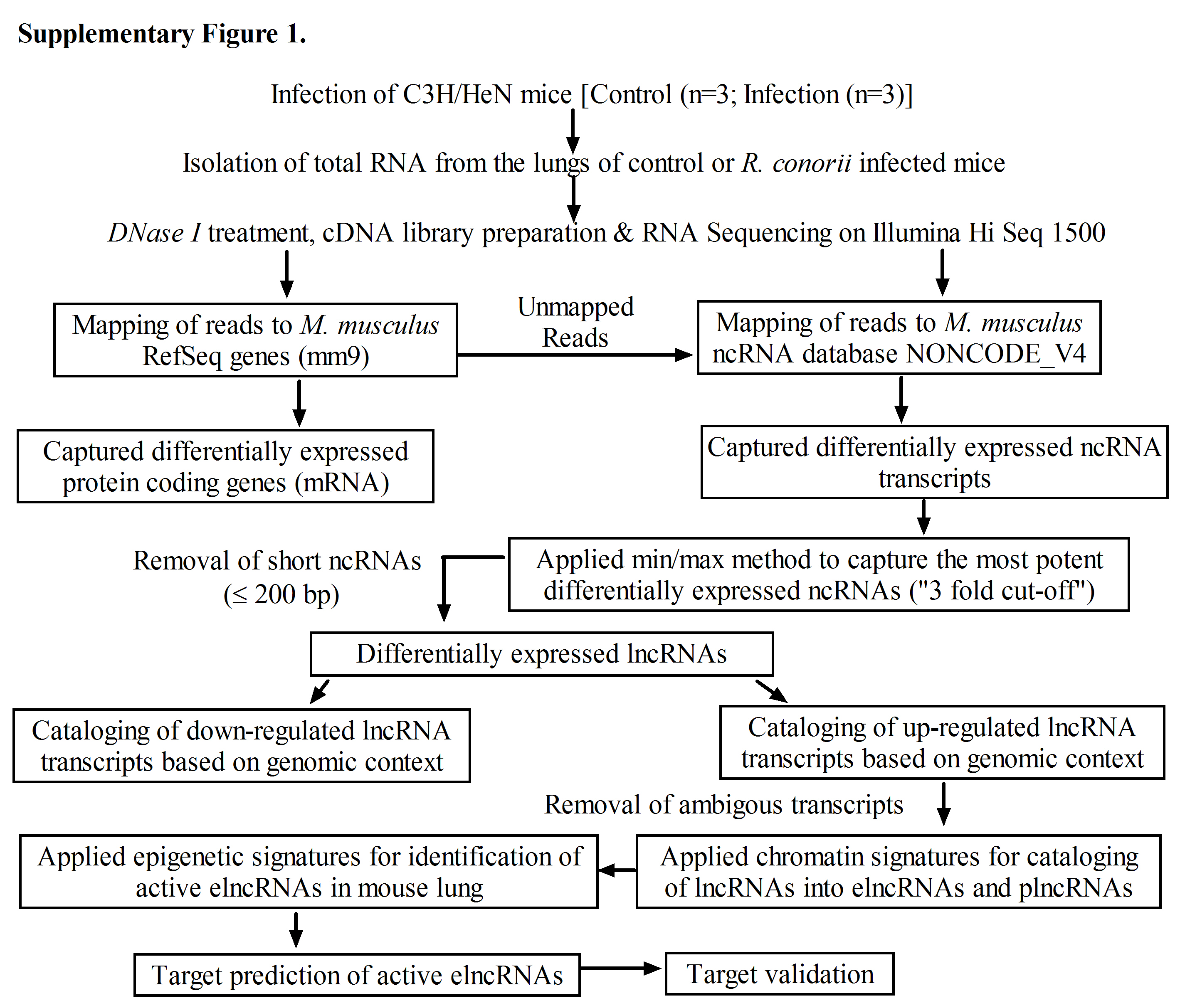

Supplement: Supplementary Figure 1 — Schematic representation of RNA-sequencing and downstream analysis. [file Image_1.JPEG]

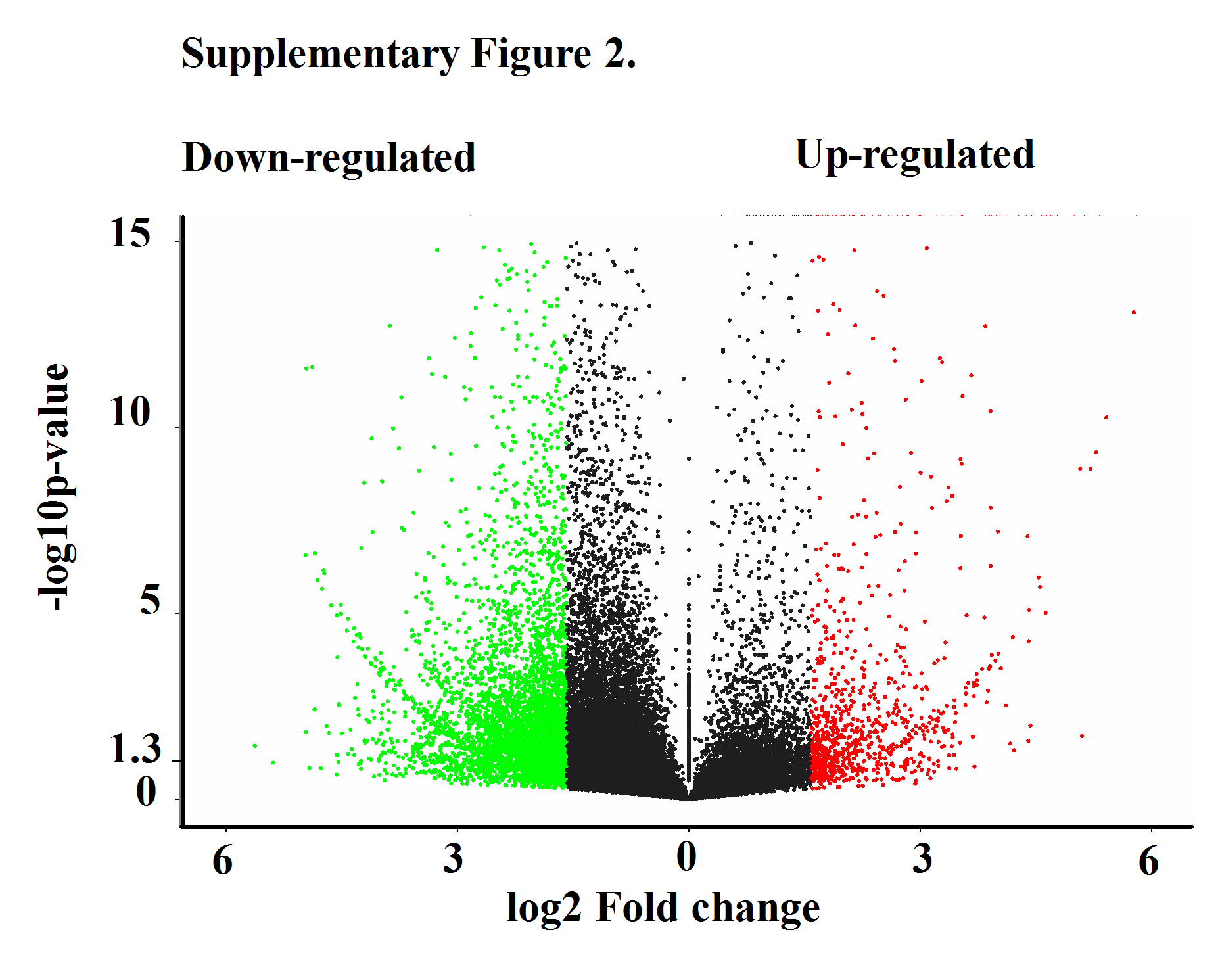

Supplement: Supplementary Figure 2 — Volcano plot displaying differentially expressed lncRNA transcripts (cut off value 3-fold, P-value -Log10 ≤ 0.05 ~ 1.3). Red and green dots on the x-axis represent positively and negatively regulated transcripts, respectively. Values on the y-axis represent the level of significance. [file Image_2.JPEG]

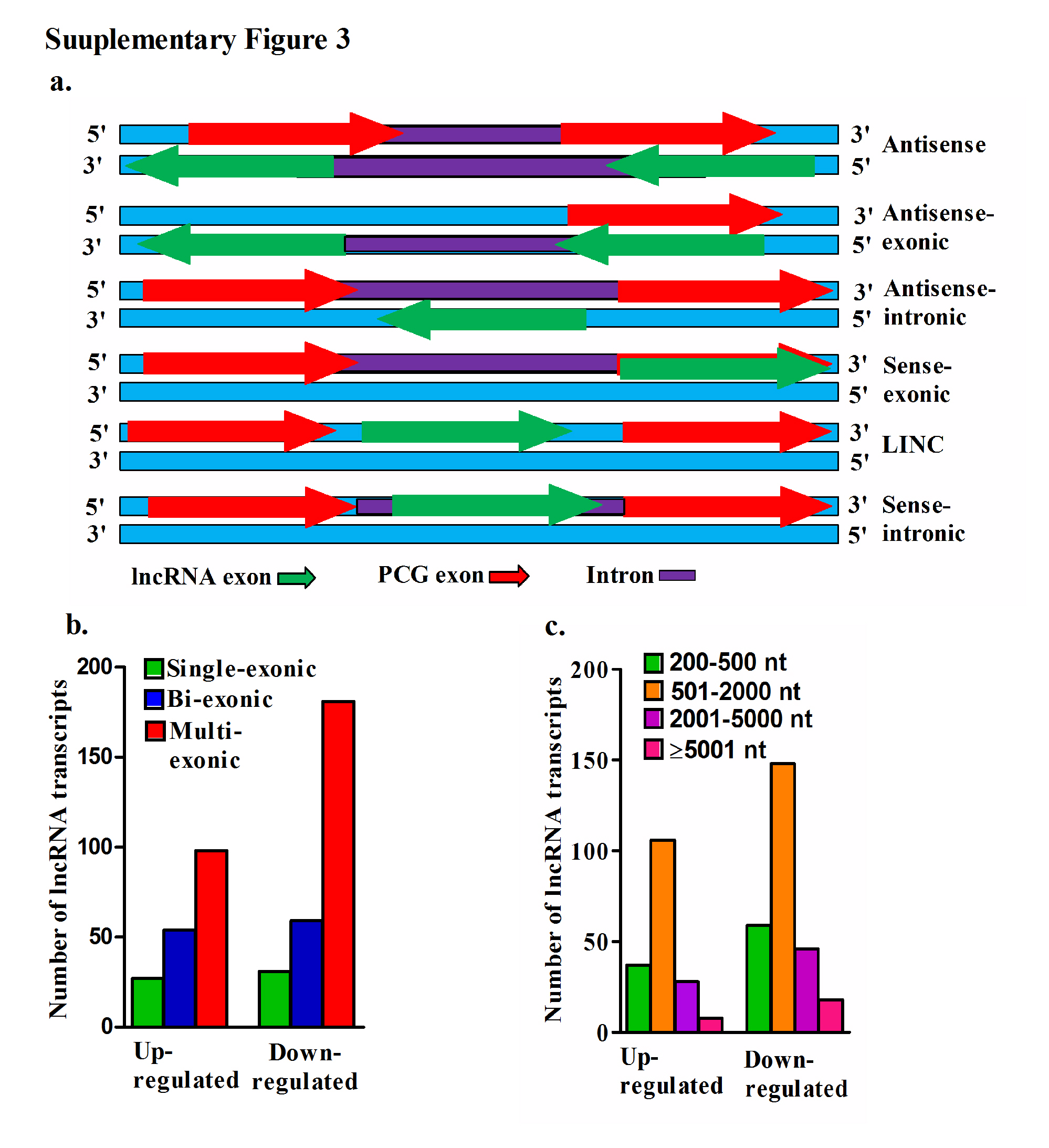

Supplement: Supplementary Figure 3 — (A) Strategies applied for cataloging of differentially expressed lncRNA transcripts based on their origin; (B) Cataloging of lncRNA transcripts based on their exon numbers; and (C) Cataloging of lncRNA transcripts based on their lengths. [file Image_3.JPEG]

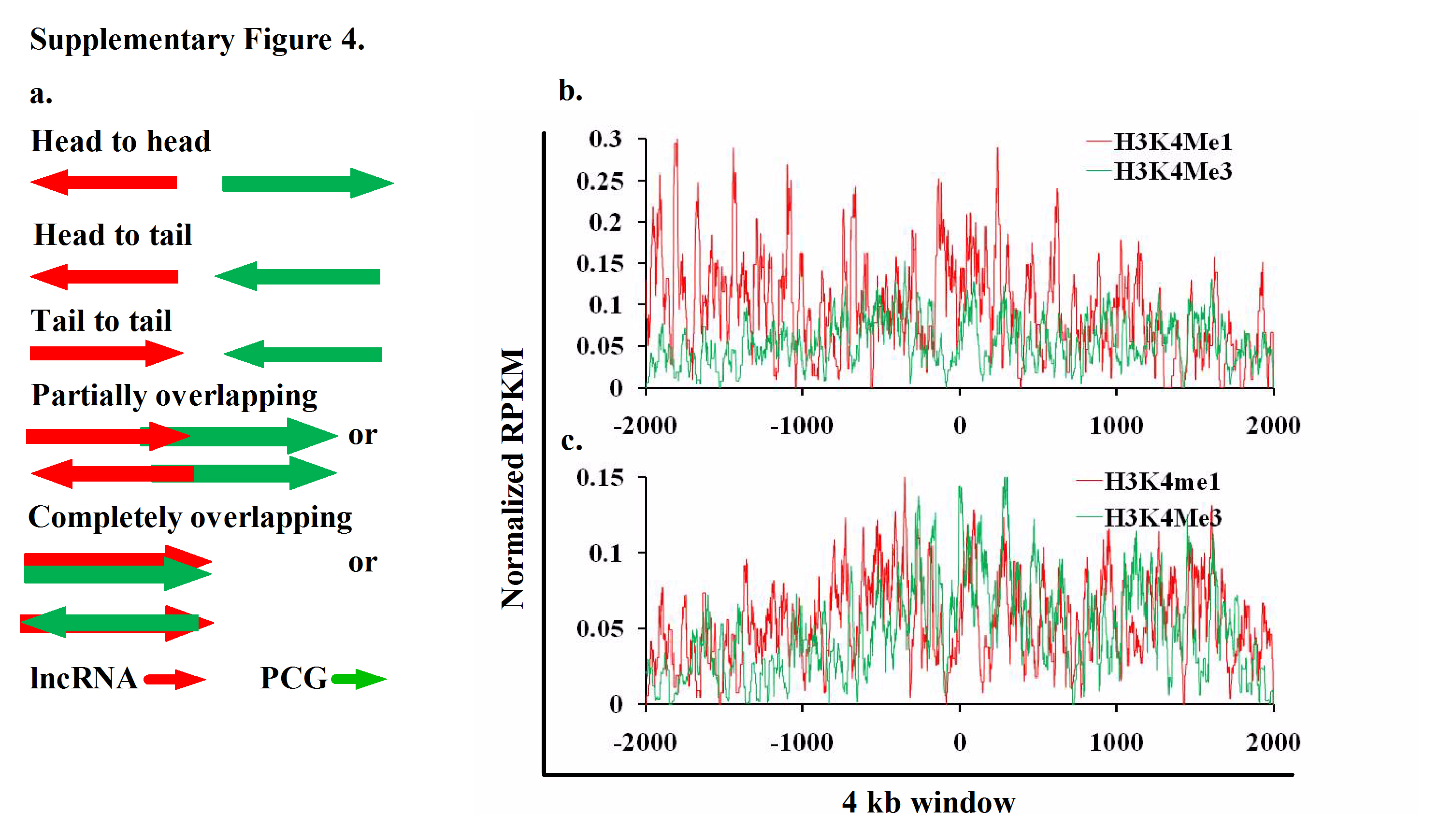

Supplement: Supplementary Figure 4 — (A) Strategies applied to identify orientation of up-regulated lncRNA transcripts with their nearby protein coding genes (PCGs); (B) Histogram depicting density of average normalized RPKM (reads per kilobase million) values of H3K4Me1 in elncRNAs and plncRNAs; and (C) Histogram depicting density of average normalized RPKM (reads per kilobase million) values of H3K4Me3 in elncRNA and plncRNAs around 4 kb window of transcription start site. [file Image_4.JPEG]

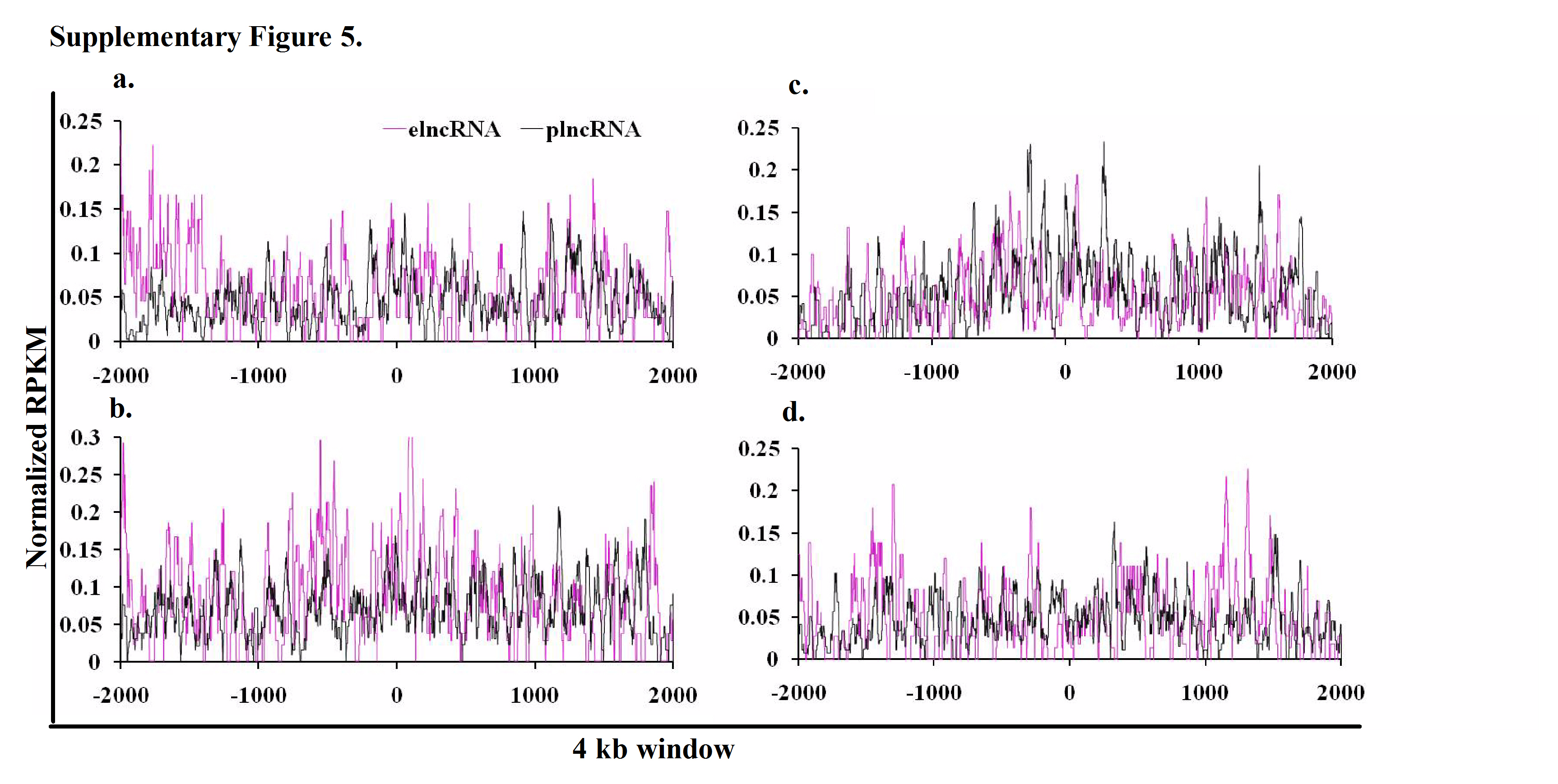

Supplement: Supplementary Figure 5 — Histogram depicting density of average normalized RPKM (reads per kilobase million) values of (A) RNA PolII; (B) p300; (C) DNaseI hypersensitivity site; and (D) CCCTC binding factor (CTCF) around 4 kb window of transcription start site of elncRNAs and plncRNAs, respectively. [file Image_5.JPEG]

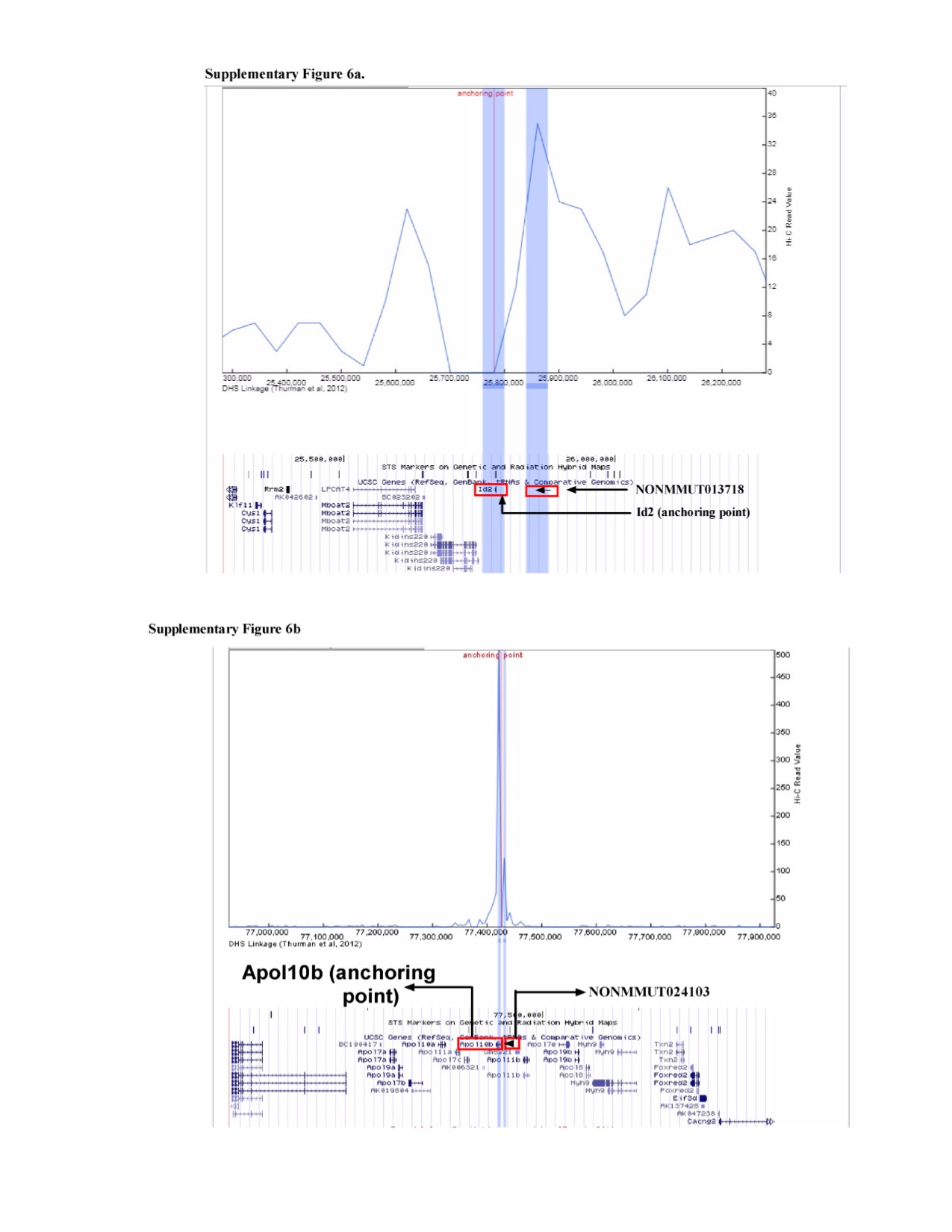

Supplement: Supplementary Figure 6 — Virtual 4C plot of (A) NONMMUT013718 and (B) NONMMUT024103 elncRNA. The anchoring point (red line) marks the position of (A) Id2 and (B) Apol10b, respectively. The contact loci are shown with blue line. [file Image_6.jpg]

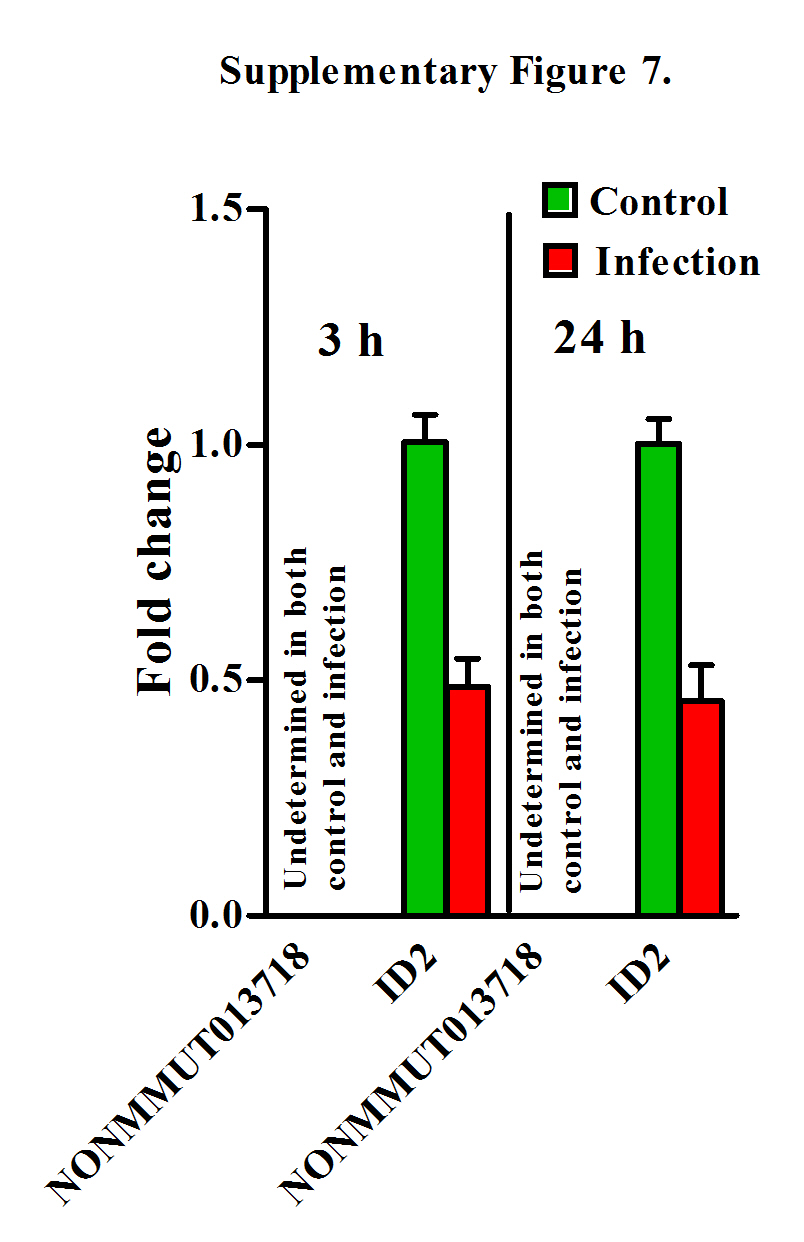

Supplement: Supplementary Figure 7 — Expression of elncRNAs and their targets in an in vitro model of R. conorii infection. Expression of NONMMUT013718 and its target Id2 in SVECs at 3 and 24 h post-infection. [file Image_7.jpg]
